# Peak amplitude of the normalized power spectrum of the electromyogram of the uterus in the low frequency band is an effective predictor of premature birth

**DOI:** 10.1371/journal.pone.0308797

**Published:** 2024-09-12

**Authors:** Žiga Pirnar, Franc Jager, Ksenija Geršak

**Affiliations:** 1 Department of Multimedia, Laboratory for Biomedical Computer Systems and Imaging, Faculty of Computer and Information Science, University of Ljubljana, Ljubljana, Slovenia; 2 Department of Obstetrics and Gynecology, Faculty of Medicine, University of Ljubljana, Ljubljana, Slovenia; 3 Department of Perinatology, Division of Obstetrics and Gynecology, University Medical Center Ljubljana, Ljubljana, Slovenia; Jordan University of Science and Technology Faculty of Computer and Information Technology, JORDAN

## Abstract

The current trends in the development of methods for non-invasive prediction of premature birth based on the electromyogram of the uterus, i.e., electrohysterogram (EHG), suggest an ever-increasing use of large number of features, complex models, and deep learning approaches. These “black-box” approaches rarely provide insights into the underlying physiological mechanisms and are not easily explainable, which may prevent their use in clinical practice. Alternatively, simple methods using meaningful features, preferably using a single feature (biomarker), are highly desirable for assessing the danger of premature birth. To identify suitable biomarker candidates, we performed feature selection using the stabilized sequential-forward feature-selection method employing learning and validation sets, and using multiple standard classifiers and multiple sets of the most widely used features derived from EHG signals. The most promising single feature to classify between premature EHG records and EHG records of all other term delivery modes evaluated on the test sets appears to be Peak Amplitude of the normalized power spectrum (*PA*) of the EHG signal in the low frequency band (0.125-0.575 Hz) which closely matches the known Fast Wave Low (FWL) frequency band. For classification of EHG records of the publicly available TPEHG DB, TPEHGT DS, and ICEHG DS databases, using the Partition-Synthesis evaluation technique, the proposed single feature, *PA*, achieved Classification Accuracy (*CA*) of 76.5% (*AUC* of 0.81). In combination with the second most promising feature, Median Frequency (*MF*) of the power spectrum in the frequency band above 1.0 Hz, which relates to the maternal resting heart rate, *CA* increased to 78.0% (*AUC* of 0.86). The developed method in this study for the prediction of premature birth outperforms single-feature and many multi-feature methods based on the EHG, and existing non-invasive chemical and molecular biomarkers. The developed method is fully automatic, simple, and the two proposed features are explainable.

## Introduction

Premature birth, defined as babies born alive before 37 weeks of gestation are complete, is a significant public health concern worldwide. According to the World Health Organization (WHO), an estimated 15 million babies are born prematurely each year, which represents about 10 percent of all live births worldwide [1]. Premature birth and its associated complications are the leading cause of mortality among neonates and children under five years old [2].

Nearly half of premature births occur after spontaneous start of labor with intact membranes, while the remaining half occur due to preterm premature rupture of the membranes, as well as labor induction or cesarean delivery for maternal or fetal indications [3, 4]. However, for the majority of cases, the cause is not known and the pathogenesis of premature labor is still poorly understood [3]. There are several treatments and preventive strategies available to reduce the risk of premature birth, however, for timely and definitive indication of treatment, clinicians must first identify, or diagnose, the danger of premature birth as soon as possible.

In clinical practice, various techniques are employed for assessing the danger of premature birth and detecting the onset of premature labor. These commonly include monitoring of the uterine dynamics using tocodynamometry [5–7], Bishop score evaluation [7–9], using molecular biomarkers like fetal fibronectin [10–13] and Interleukin-6 (IL-6) [6, 13–15], and assessing known risk factors [16–18]. However, tocodynamometry and Bishop score have been shown to be unreliable predictors of premature labor [6], while there is ample evidence to suggest that methods for predicting premature birth based on fetal fibronectin or IL-6 suffer from low sensitivities [19–21]. Furthermore, a short cervical length is a well known risk factor for premature birth [3, 22, 23], but it suffers from low sensitivity in low-risk pregnancies [9, 24–26]. As such, these methods may be more suited to identify which of the high-risk pregnancies are not at risk for premature birth, due to their high specificity. Moreover, several additional risk factors are known to be associated with spontaneous premature birth, including history of premature birth [27], multiple gestations [28], short inter-pregnancy interval [29], prior conization of the cervix [30], uterine anomalies [31], diabetes and hypertension [28, 32], as well as substance abuse, multiple socioeconomic conditions, and lifestyle factors [33, 34]. However, the prediction of premature birth based solely on these risk factors is uncertain.

Researchers have also studied the use of novel chemical and molecular biomarkers for the prediction of premature birth prior to labor onset [35]. These prediction models either use a single chemical, gene, protein, or microRNA biomarker (i.e. a single feature), or are based on multiple biomarkers (i.e. multiple features). Table 1 shows an overview of some of the most recent studies of novel genomic, proteomic, and transcriptomic biomarkers for non-invasive prediction of premature birth that reported the achieved Area Under Curve (*AUC*), i.e. the area under the Receiver Operating Characteristic (ROC) curve. The first group of methods in Table 1 used a single feature [36–39], while the second group used multiple features [40–44]. If a single feature is used, the best performing models, among those listed in Table 1, include the following single biomarkers for premature birth: insulin-like growth factor binding protein 3 (IGFBP-3) [36], and microphage migration inhibitory factor (MIF) [37], with the *AUC* of 0.79 and 0.71, respectively.

**Table 1:** An overview of some of the most recent methods for non-invasive prediction of premature birth based on chemical and molecular biomarkers.

Study	Year	Biomarker(s) (Model)	Features	*AUC*	Description and comments
[36]	2016	IGFBP-3 (LogR)	1	0.79	Maternal serum concentration of insulin-like growth factor binding protein 3 (IGFBP-3) (selected out of 14 candidate biomarkers).
[37]	2018	MIF factor (LogR)	1	0.71	Maternal plasma concentrations of macrophage migration inhibitory (MIF) factor. Noteworthy, MIF showed significantly greater discriminatory ability compared to C-reactive protein (*AUC* 0.61) and interleukin 6 (*AUC* 0.63).
[38]	2017	Protein PROK1 (ROC)	1	0.69	Study evaluated several plasma proteins. Best *AUC* was achieved using prokineticin-1 (PROK1).
[39]	2021	microRNA miR4695-5p (ROC)	1	0.65	The expression levels of miR200a and miR4695-5p microRNA in serum were evaluated using RT-PCR. Best *AUC* was achieved with miR4695-5p.
[40]	2021	Gene expression (ROC)	3	0.82	Best *AUC* was achieved using B2M, RUNX3, and TLR4 (nitric oxide pathway-related) gene expression.
[41]	2020	Blood microRNAs (ROC)	12	0.80	Best *AUC* was achieved using top 12 microRNAs (based on the *p*-values obtained using the training set) out of 45 that were initially chosen from a pool of 2,550 microRNAs.
[42]	2023	PAPP-A, known risk factors, and Cervix Length (LogR)	5	0.79	Best *AUC* was achieved using pregnancy-associated plasma protein-A (PAPP-A) multiple of the median, parity, conization, history of premature birth, and cervix length.
[43]	2021	Plasma proteins (RF)	50	0.76	Top 50 proteins selected by RF model importance from panel of 1,125 proteins. Plasma proteomics outperformed whole-blood transcriptomics (*AUC* 0.62).
[44]	2023	Cervical microRNAs (SVM)	10	0.71	Top 10 cervical microRNA selected out of 754 microRNAs on the PCR array using a relief-based feature selection.

The upper and lower sections of the table include the methods based on a single feature (biomarker) and the methods based on multiple features, respectively. For each method, the number of features and the achieved Area Under the ROC Curve (*AUC*) are shown in the table. LogR—Logistic Regression, ROC—The cut-off value was determined using ROC curve analysis, RF—Random Forest, SVM—Support Vector Machine.

Notably, all of the methods based on a single biomarker listed in Table 1 exhibit *AUC* scores below 0.80. As such, there is an increasing tendency towards the use of more complex models with multiple biomarkers which may decrease their ease of use, diminish their explainability, increase associated costs related to sample collection and analysis, and thereby potentially decrease or prevent their use in a clinical setting. Among the most recent methods based on multiple biomarkers in the bottom half of Table 1, the method yielding the highest *AUC* (0.82) was based on the expression levels of three genes (B2M, RUNX3, and TLR4) [40]. The next best performing method in Table 1 relied on the expression levels of the top 12 microRNAs in maternal peripheral blood, yielding the *AUC* of 0.80 [41]. Despite their increased complexity, these two methods yielded only modest improvements in performance over methods based on a single biomarker, in terms of the *AUC*.

### Uterine electromyogram

The uterine electromyogram, recorded from the abdominal wall of a pregnant woman, also known as the electrohysterogram (EHG), has shown great potential as a cost-effective and non-invasive diagnostic tool for assessing the danger of premature birth [5, 7, 45–47]. The uterus is a highly complex non-linear dynamic system comprising billions of interconnected smooth myocyte cells which produce measurable changes in electrical potentials, reflecting the electrical activity of the uterus [48, 49]. Traditionally, the EHG was employed for non-invasive quantitative assessment of uterine contractions [45, 46, 48–50]. However, despite decades of research, the underlying physiological processes that lead to premature birth and the precise electrophysiological mechanisms driving EHG generation remain poorly understood. Nevertheless, studies have shown that the EHG appears to contain sufficient information to enable efficient non-invasive prediction of premature birth several weeks, or even months, before delivery [5, 51].

To predict premature birth using the EHG, researchers focus on two approaches. The first approach tries to differentiate between the non-labor and labor phases (which usually translates into recognition of contractions related to imminent delivery or prediction of delivery time) [5, 7, 47, 52–56]. The second, increasingly more common approach attempts to differentiate between premature EHG records obtained from pregnancies that eventually end in premature delivery (before the 37th week of gestation) and term EHG records obtained from pregnancies that eventually reach term delivery (during the 37th week of gestation or later) [51, 57, 58]. In both cases, under the traditional feature engineering approach, the features (parameters) to assess the relevant underlying physiological mechanisms are extracted from the EHG to identify the approaching labor phase, or to distinguish between premature and term records. The features may be extracted from segments of the EHG containing individual contraction burst intervals (contraction intervals), or from whole signals of individual EHG records without the need to first detect contractions and isolate, or annotate, individual contraction intervals. Moreover, there is strong evidence to suggest that segments between contraction intervals, i.e. non-contraction intervals, also known as dummy intervals, contain sufficient information to effectively separate between premature and term EHG records [57].

Various linear and non-linear signal processing techniques have been used to extract the features from the EHG, however, it is well established that the latter are better suited for assessing the underlying non-linear physiological processes [51]. The spectral and non-linear features extracted from the EHG commonly include the Peak Frequency (*PF*) and Median Frequency (*MF*) of the power spectrum [59], Peak Amplitude of the normalized power spectrum (*PA*) [57], and entropy measures like the widely used Sample Entropy (*SE*) [57, 59, 60] and approximate entropy [60–62]. Most notably, features *PF*, *MF*, and *PA* are easily understandable and suitable for directly and transparently assessing the shifts and intensity of the frequency content of the EHG in the frequency bands corresponding to background physiological mechanisms. On the other hand, *SE* is used to estimate the level of regularity, or predictability, of the signal in the selected frequency bands, thus assessing the presence or absence of underlying periodic physiological mechanisms. Moreover, a multitude of more complex and intricate entropy measures such as dispersion entropy [63, 64], bubble entropy [63, 65], fuzzy entropy [66, 67], permutation and phase entropy [67], and multivariate multiscale fuzzy entropy [68] have shown promise, yet their increased complexity may diminish their applicability and interpretability.

This proliferation of features seems to have become pronounced with the introduction of the Term-Preterm EHG Database (TPEHG DB) [59, 69] which spurred a rapid progression in the development of methods for the prediction of premature birth using various signal processing techniques and classification approaches with entire EHG records and non-linear features. The TPEHG DB includes 262 term and 38 premature EHG records, recorded early (around the 23rd week of pregnancy) or later (around the 31st week of pregnancy). However, due to the imbalance between premature and term records in this database, many researchers resorted to using over-sampling to synthesize new samples and balance the classes prior to partitioning the data into the training and test sets (Synthesis-Partition evaluation technique). For many of these methods, researchers reported spectacular classification performance results, however, a subsequent study [70] revealed that applying over-sampling before partitioning constituted a significant methodological flaw, resulting in data leakage and excessively optimistic classification performance results. Moreover, the authors re-evaluated a variety of related methods, by correctly applying over-sampling only after partitioning the data (Partition-Synthesis evaluation technique), and showed that the realistic *AUC* scores of those methods were closer to 0.65 [70].

The introduction of Term-Preterm EHG DataSet with Tocogram (TPEHGT DS) [57, 69] has obviated the need for over-sampling when evaluating the classification performance of methods for the prediction of premature birth, as it includes a balanced set of 13 premature and 13 term EHG records. Furthermore, premature records contain 47+47 annotated contraction and dummy intervals, while term records contain 53+53 annotated contraction and dummy intervals. Therefore, even in the case of classifying between premature and term intervals (instead of using whole signals), balancing may not be required since any balancing will have very little effect on the evaluation since the number of premature and term intervals is almost equal. However, because individual annotated intervals do not represent independent samples, and due to the small number of records in the TPEHGT DS, evaluation of methods using only this dataset may not be reliable. Additionally, it is important to note that methods utilizing annotated intervals from the TPEHGT DS may not be considered fully automated. These intervals were manually annotated by experts, first by examining the tocogram signal (which measures the mechanical uterine activity using an external tocodynamometer), and then looking for the accompanying characteristics of the EHG signals [57].

Due to the compatibility between the records of the TPEHG DB and TPEHT DS (in terms of recording protocol and recording device), several researchers have also combined the EHG records from both datasets. This was usually done to increase the number of available premature records, yet this once again introduced the challenge of dealing with an imbalanced dataset. To assess the classification performance using the records of the combined dataset, early studies [63, 71] initially employed the Synthesis-Partition evaluation technique, while recent studies [67, 72] utilizing the combined dataset have shifted towards using the recommended Partition-Synthesis evaluation technique [70].

#### Single-feature EHG methods

Table 2 shows an overview of some of the most recent methods for the prediction of premature birth, or classification between premature and term records, based on a single feature extracted from the EHG. The studies in Table 2 are grouped according to the used dataset(s). The first group includes studies [57, 73] that used annotated dummy intervals of the TPEHGT DS, the second includes studies [58, 74] that used closed datasets, while only one single-feature study [75] used records of the TPEHG DB and the Partition-Synthesis evaluation technique (due to the class imbalance). Notably, none of the single-feature studies in Table 2 used a combined dataset with premature and term records of the TPEHG DB and TPEHGT DS. The most promising single-feature method [73] yielded the highest *AUC* of 0.85, using *SE* extracted from signal S2 of annotated dummy intervals of the TPEHGT DS in the frequency band B2 (2.2-3.5 Hz) [57]. Next, the method reported in [57] achieved the *AUC* of 0.75 using *PA* of signal S2 extracted from annotated dummy intervals of the TPEHGT DS in the frequency band B1 (1.0-2.2 Hz) [57]. However, these results may be less realistic since both studies [57, 73] classified between annotated intervals from only 13 premature and 13 term records of the TPEHGT DS, and neither method is fully automated because they require prior annotation of non-contraction (dummy) intervals.

**Table 2:** An overview of some of the most recent methods for prediction of premature birth based on the EHG based a single feature (biomarker). The studies are grouped according to the used dataset.

Study	Year	Model	Dataset(s)	Features	*CA* [%]	*AUC*	Description and comments
[73]	2020	QDA	TPEHGT DS (dummy intervals)	1	84.0	0.86	Best *AUC* was achieved with sample entropy for signal S2 in band B2 (2.2-3.5 Hz) of annotated non-contraction (dummy) intervals.
[57]	2018	QDA	TPEHGT DS (dummy intervals)	1	69.81	0.75	If using a single feature, best *AUC* was achieved with peak amplitude of the normalized power spectrum for signal S2 in band B1 (1.0-2.2 Hz) of annotated non-contraction (dummy) intervals.
[58]	2018	ROC	Closed dataset (contraction intervals)	1	75	0.75	Closed dataset with 58 EHG records from women admitted for premature contractions. Final dataset for ROC analysis included 804 annotated contraction intervals. Best *AUC* was achieved using modified approximate entropy for signal E2 in the frequency band 0.3-0.8 Hz.
[74]	2022	ROC	Closed dataset (whole signals)	1	-	0.72	Closed dataset with 72 EHG records collected around the 34th week of gestation. The studied feature was mean uterine activity, expressed in microvolts/epoch.
[75]	2022	Bayes	Early and Later TPEHG DB (whole signals)	1	59	0.65	If using a single feature, extracted from whole signals in the time-domain, best *AUC* was achieved with approximate entropy for signal S3 (unfiltered).

For each method, the achieved Classification Accuracy (*CA*) and the Area Under the ROC Curve (*AUC*) are shown in the table, if reported in the respective studies. Models: ROC—The cut-off value was determined using ROC curve analysis, QDA—Quadratic Discriminant Analysis, Bayes—Naive Bayes Classifier.

#### Multiple-feature EHG methods

Table 3 shows an overview of some of the most recent methods based on multiple features extracted from the EHG. The studies are grouped according to the used datasets: studies [57, 73, 76, 77] that used only annotated intervals of the TPEHGT DS, studies [75, 78, 79] that used the records of the TPEHG DB, and studies [67, 72, 80] that used the combined dataset comprising records of both the TPEHG DB and TPEHGT DS. Note that methods which were evaluated using the Synthesis-Partition evaluation technique were excluded due to overly optimistic results according to [70].

**Table 3:** An overview of some of the most recent methods for prediction of premature birth based on the EHG based on multiple features. The studies are grouped according to the used signal scope (intervals or whole signals) and dataset.

Study	Year	Model	Dataset(s)	Features	*CA* [%]	*AUC*	Description and comments
[76]	2020	QDA	TPEHGT DS (dummy and contraction intervals)	19	91	0.97	Feature selection was performed using the whole dataset which may have led to overfitting. Best *AUC* was achieved using coupling strength and directionality indices, derived from Granger causal analysis, for contraction and dummy intervals of EHG and tocogram signals.
[77]	2023	SVM	TPEHGT DS (dummy intervals)	9	87.1	-	*AUC* was not reported. Highest *CA* was achieved using decision fusion of the output from three classifiers with three Hjorth parameters for each signal (i.e. nine features).
[73]	2020	QDA	TPEHGT DS (dummy intervals)	2	85.8	0.89	If using two features, best *AUC* was achieved with sample entropy in band B2 (2.2-3.5 Hz) for signals S2 (feature 1) and S3 (feature 2) of annotated non-contraction (dummy) intervals.
[57]	2018	QDA	TPEHGT DS (dummy intervals)	2	70.75	0.80	If using two features, best *AUC* was achieved with peak amplitude of the normalized power spectrum in band B1 (1.0-2.2 Hz) for signal S2 (feature 1) and for tocogram (feature 2) of annotated non-contraction (dummy) intervals.
[78]	2022	HVG + SVM	Early and Later TPEHG DB (whole signals)	>200	91	0.97	Feature selection (based on Fisher’s discriminate analysis and F-score) was performed using the whole dataset which may have led to overfitting. Without feature selection, the *AUC* was 0.70. Furthermore, HVG-based features are complex and not easily understandable.
[75]	2022	Bayes	Early and Later TPEHG DB (whole signals)	8	75	0.84	Feature selection (based on Principal Component Analysis) was performed using the whole dataset which may have led to overfitting. Best *AUC* was achieved using sample entropy, extracted from eight out of 41 time-frequency series of the short-time Fourier transform.
[79]	2023	LSTM or CNN	Early and Later TPEHG DB (whole signals)	12	-	0.58	Complex deep learning model. Based on the LSTM or CNN, combined with a fully connected layer, using time series of sample entropy, median frequency, and peak frequency, extracted from 50 intervals of signals S1, S2, and S3 in the frequency band 0.34-1.0 Hz. Best *AUC* was achieved with sample entropy and clinical data.
[72]	2022	LDA	Early and Later TPEHG DB and TPEHGT DS (whole signals)	58	-	0.95	Complex set of features. Top features were selected out of 227 (temporal, spectral, and non-linear) features and obstetric parameters using the genetic algorithm. The optimal subset of features was not reported.
[67]	2023	SVM	Later TPEHG DB and TPEHGT DS (two minute intervals)	7	88.52	0.89	Results may be overly optimistic as the final test set comprised highly correlated overlapping intervals (each treated as if it were from a separate pregnancy) extracted from only four term and four premature (pre-selected) records. Also, under-sampling of the term class was performed only once (no repetitions were performed).
[80]	2023	BiLSTM + LogR	Later TPEHG DB and TPEHGT DS (whole signals)	>600	-	0.78	Complex deep learning model. Signal S1 was transformed using 1200-point short-time Fourier transform. The resulting 600 time-frequency series were used as input to the BiLSTM. Best *AUC* was achieved in combination with clinical data (eight obstetric parameters).

For each method, the achieved Classification Accuracy (*CA*) and the Area Under the ROC Curve (*AUC*) are shown in the table, if reported in the respective studies. Models: QDA—Quadratic Discriminant Analysis, Bayes—Naive Bayes Classifier, HVG—Horizontal Visibility Graph, SVM—Support Vector Machine, LDA—Linear Discriminant Analysis, BiLSTM—Bidirectional Long Short-Term Memory, LogR—Logistic Regression LSTM—Long Short-Term Memory, CNN—Convolutional Neural Network.

In Table 3, the method yielding the highest *AUC* of 0.97 [78] used over 200 features, extracted from the Horizontal Visibility Graph (HVG), which was constructed from whole EHG signals of the TPEHG DB in the time-frequency domain. However, this transformation of the input signals into the HVG-based latent space may completely obfuscate the feature meaning, in the physiological sense, and severely diminish the much-needed insight into the underlying physiological mechanisms. Besides, their results may be overly optimistic since feature selection was performed using the whole dataset (i.e. no validation set was used and no unseen data remained for testing), which may have led to overfitting of the final feature selection to the test sets. This was also done in [76], which used annotated contraction and dummy intervals of the TPEHGT DS and yielded the same *AUC* of 0.97. The next best performing method [72] yielded an *AUC* of 0.95, using the genetic algorithm to obtain a complex feature set with 58 (out of 227) temporal, spectral, and non-linear features as well as obstetric parameters, but the authors did not report which features ended up in the optimal subset.

Notably, very few of the multi-feature methods in Table 3 used less than 10 features. In [73], the authors also evaluated the classification performance using only two meaningful features, *SE* of signals S2 and S3 in the band B2 (2.2-3.5 Hz), yielding an *AUC* of 0.89 when using annotated dummy intervals of the TPEHGT DS. Again, it is important to note that the TPEHGT DS contains only 13 premature and 13 term records, and the reported results may be less reliable, since the intervals used for training and testing may come from the same EHG records. Besides, this method is not fully automated, as it depends on using manually annotated intervals. Moreover, annotating contraction intervals or dummy (non-contraction) intervals would be extremely time-consuming and subjective in clinical practice, and therefore less reliable. Furthermore, for the method reported in [67], the best *AUC* of 0.89 was achieved using seven features, however, their results may be overly optimistic due to having used a single under-sampling of the term class with no repetitions.

Two of the most recent methods [79, 80] utilized complex deep learning models. However, because little to no effort was put into their explainability, these models represent complex “black-box” prediction machines which provide little to no insight into the underlying physiological mechanisms. In particular, in [80] the authors used more than 600 features, extracted from whole later EHG records of the TPEHG DB and TPEHGT DS, with the Bidirectional Long-Short Term Memory (BiLSTM) and logistic regression, yielding the *AUC* of 0.78. In the second study [79], the authors used 12 time-series of feature values (*SE*, *MF*, and *PF*), extracted from sequential overlapping segments of signals S1, S2, and S3 of EHG records of the TPEHG DB, with the Long-Short Term Memory (LSTM) or Convolutional Neural Network (CNN), yielding the *AUC* of only 0.58. Notably, neither of these two “black-box” approaches utilizing complex deep-learning based methods yielded higher *AUC* scores than those yielded by more traditional methods based on feature engineering.

#### Non-spontaneous delivery modes

The datasets used by the related studies only include EHG records from premature and term pregnancies that ended spontaneously. However, it is entirely probable that pregnancies end in other common non-spontaneous delivery modes like induced labor or cesarean section. In our previous study [81], we quantitatively characterized and assessed the separability of the sets of induced, cesarean, and induced-cesarean EHG records of the recently introduced Induced-Cesarean EHG DataSet (ICEHG DS) [69, 82, 83], alongside premature and term spontaneous EHG records of the TPEHG DB and TPEHGT DS, using several of the most widely used non-linear features extracted from the EHG signals. Additionally, the study proposed several new bands for feature extraction, and found that *PA* in the newly proposed low frequency band, denoted as B0L’ (0.125-0.575 Hz) [81], which approximately matches the known Fast Wave Low (FWL) frequency band (0.1-0.6 Hz) according to [49], is a powerful feature to separate between later premature and term records, regardless of delivery mode. However, the previous study [81] did not evaluate the classification power of the proposed features, nor quantitatively assess whether the occurrence of common non-spontaneous delivery modes affects the classification power of the proposed features.

### The aims of this study

There is an increasing tendency towards the use overly complex methods and less understandable features which lack explainability and hardly provide any new insights into the underlying physiological mechanisms, even though the causes of premature birth are still poorly understood. Furthermore, to our knowledge, no prior studies assessed whether non-spontaneous delivery modes impact the classification performance of the methods for the prediction of premature birth based on the EHG. However, a robust approach to accurate prediction of premature birth should take into account that pregnancies may also end in other common term delivery modes like induced delivery and cesarean section.

The aims:

1 To identify the most promising features, derived from the EHG signals and their power spectra in the most widely used and recently investigated frequency bands below and above 1.0 Hz, as candidate biomarkers for assessing the danger of premature birth using the sequential forward selection of features method, stabilized with the frequency-based aggregation of the selected features technique.
2 To assess the classification power of the identified most promising features, using later recorded premature and term EHG records from the publicly available term-preterm EHG datasets (TPEHG DB, TPEHGT DS, and ICEHG DS), and a variety of traditional classifiers. Due to the imbalance between the number of available premature and term records, the classification performance was evaluated using the Partition-Synthesis evaluation technique.
3 To develop a simple and explainable method for efficient, non-invasive, and fully automated prediction of premature birth based on the EHG, using one or at most two of the identified most promising features (biomarkers).
4 To quantitatively assess the impact of common term non-spontaneous delivery modes (induced delivery, cesarean section) on the predictability of premature birth when using the developed method and the enriched representation of the term class with records from the recently introduced and publicly available ICEHG DS.

In this study, Peak Amplitude of the normalized power spectrum (*PA*), extracted from EHG signals in the low frequency band B0L’ (0.125-0.575 Hz), related to the known FWL frequency band, and Median Frequency (*MF*) of the power spectrum in the band B3 (3.5-5.0 Hz), related to the influence of the maternal heart on the uterus, were identified as the most promising features to classify between later premature and term records of the TPEHG DB, TPEHGT DS, and ICEHG DS. Furthermore, the addition of records of non-spontaneous delivery modes from the ICEHG DB to the term class did not reduce the classification performance, suggesting that these potential delivery outcomes do not significantly impact the power of the proposed method to assess the danger of premature birth. The proposed method for the prediction of premature birth is effective, simple, explainable, robust, fully automated, and appears to outperform existing single-feature and many multi-feature methods based on the EHG, as well as existing non-invasive chemical and molecular biomarkers.

## Materials and methods

In the present study, we classified between later premature and term records of several publicly available EHG datasets using several features extracted from the EHG signals in various frequency bands. The most promising features to be candidate biomarkers were first identified using feature selection based on the Sequential Forward Selection (SFS) method [84], stabilized with the frequency-based aggregation of the selected features technique [85], employing a variety of traditional classifiers and various feature sets. The classification performance of the identified most promising features was then evaluated using the selected traditional classifiers. Furthermore, in addressing the class imbalance, we strictly adhered to the Partition-Synthesis evaluation technique [70], i.e. over-sampling was performed only after partitioning the dataset into training and test sets.

### EHG datasets and groups of records

For feature selection and classification, we used EHG records from the Term-Preterm EHG Database (TPEHG DB) [59, 69], Term-Preterm EHG DataSet with Tocogram (TPEHGT DS) [57, 69], and Induced-Cesarean EHG DataSet (ICEHG DS) [69, 82, 83]. The records of all three datasets are fully compatible in terms of electrode placement (E1, E2, E3, E4), signal definitions (S1, S2, and S3), sampling frequency (20 Hz), recording duration (around 30 minutes) and recording apparatus. The tocogram signal is present only in records of the TPEHGT DS, hence, it was not used in our study to ensure the consistency of the features extracted from signals available in all three datasets used in this study. Likewise, we did not use any additional clinical parameters included in the EHG records. Common clinical parameters included in the EHG records from these three datasets include maternal age, parity, number of abortions, maternal weight, placental position, and smoker status. Unfortunately, some of these were missing in several EHG records used in this study, and thus were not considered as potential features in our analysis. Besides, the results of two recent studies [79, 80] suggest that this clinical data only has a modest impact on the predictability of premature birth (in both studies, the addition of a multitude of clinical parameters improved the AUC score only by 0.04).

The TPEHG DB contains 300 EHG records, of those 19 premature and 143 term records were collected early in the pregnancy, around the 23rd week of gestation (early records), and 19 premature and 119 term records were collected later in the pregnancy, around the 31st week of gestation (later records). The TPEHGT DS contains 13 premature and 13 term records, all collected later in the pregnancy (again, around the 31st week of gestation). As such, similarly to recent related studies [67, 80], we used only later records of both dataset for consistency in terms of the time when the records were collected. Besides, the results of our previous study [81] show that features exhibit significantly different powers to separate between premature and term records when recorded early or later in the pregnancy.

The later premature records of the TPEHG DB and the TPEHGT DS were merged into group PL (19+13 = 32 Premature Later records), and the later term records of these two datasets into group TL (119+13 = 132 Term Later records). The records from groups PL and TL were initially used to identify the most promising features to distinguish between premature and term pregnancies, and to evaluate the performance of the classifiers. However, these records were collected from pregnancies with vaginal deliveries that started spontaneously. The assumption of spontaneous vaginal delivery mode at the time of collecting the EHG record for the purposes of predicting premature birth is unrealistic, since the pregnancy may end in other common delivery modes like term induced delivery or cesarean section. Therefore, for a more realistic assessment of the performance of the proposed method for automated premature birth prediction, we also utilized the recently introduced ICEHG DS. The ICEHG DS contains 126 EHG records collected from pregnancies that ended in term vaginal delivery that did not start spontaneously, necessitating labor induction (induced records), or in term delivery by emergency cesarean section, without or with prior labor induction (cesarean and induced-cesarean records, respectively). For consistency with records of both the TPEHG DB and the TPEHGT DS in terms of gestational age at the time of recording, we used only later records of the ICEHG DS. Thus, 43 later induced (group IL), eight later cesarean (group CL), and 13 later induced-cesarean records (group ICL) were subsequently used to enrich the representation of term pregnancies, i.e. that of the term class. This allowed us to evaluate the performance of binary classifiers in distinguishing between records representing premature pregnancies (group PL) and those representing term pregnancies (groups TL, IL, CL, and ICL, together comprising (119+13)+43+8+13 = 196 records), regardless of delivery mode. More importantly, it enabled us to assess whether the inclusion of the non-spontaneous delivery modes impacts the power of the proposed method to predict premature birth.

### Extraction of features

In the traditional feature engineering approach, suitable features are first constructed to represent spectral and non-linear parameters of EHG signals within the selected frequency bands [51]. This is done to assess the underlying physiological mechanisms believed to be localized in different locally stationary frequency bands [57]. In this study, we employed various univariate spectral and non-linear features extracted from EHG signals within the selected frequency bands that were previously defined and used in [57, 59, 81].

#### Selected signals and scope of analysis

The EHG signals common to all three datasets used in this study (TPEHG DB, TPEHGT DS, and ICEHG DS) measure the difference in the electric potentials between two horizontally oriented or two vertically oriented electrodes. All three datasets include signals S1, S2, and S3. In particular, signals S1 (E2-E1) and S3 (E4-E3) are horizontally oriented, and are reflective of the electrical activity of underlying physiological mechanisms in the horizontal direction [57, 73]. Signal S2 (E2-E3) is vertically oriented, and is reflective of the electrical activity of the underlying physiological mechanisms in the vertical direction [57, 73]. In this study, we used the vertically oriented signal, S2, and only one of the two horizontally oriented signals, S3, since it measures the electrical activity closer to the cervix, compared to signal S1. Besides, in the previous study [81] we found that the features extracted from signals S1 and S3 exhibited similar powers to separate between premature and term records.

The features were extracted from whole 30-minute EHG signals, using the same feature extraction techniques and pre-processing steps reported in our previous study [81], which initially characterized the records of the ICEHG DS and assessed the saparability power of a variety of features in a variety of frequency bands. Additionally, it is important to note that using whole signals streamlines the analysis of EHG records by eliminating the need for prior annotation, or detection, of contraction intervals. This, in turn, enables fully automated non-invasive prediction of premature birth.

#### Frequency bands

The physiological processes of the uterus are reflected in the EHG across various frequency ranges and with different intensities, whereas the signal spectral content, complexity, and intensity vary as pregnancy progresses [57, 59]. The electrical activity of contractions is normally reflected in the EHG in the known Fast Wave Low (FWL), 0.1-0.6 Hz, and Fast Wave High (FWH), 0.6-3.0 Hz, frequency bands [48, 49, 51, 86, 87]. Consequently, the selected spectral and non-linear features to assess these physiological mechanisms are typically extracted from signals in the related frequency bands, usually with a lower limit of 0.08, 0.3, or 0.34 Hz, and the upper limit of 1.0 Hz [51]. The aim of using these limited bands is to separately assess the uterine mechanisms while trying to exclude the effects of frequency components of other etiologies. In particular, below 0.34 Hz, the EHG also contains overlapping frequency components related to maternal breathing [88], while above 1.0 Hz, the EHG also contains frequency components related to the maternal heart [57, 81]. In our previous characterization study [81], we assessed the power of the most widely used non-linear features extracted from several bands, and the results suggest that frequency components below 0.3 Hz and above 1.0 Hz contain valuable information for the prediction of premature birth. In the previous study, we defined two new frequency bands, B0L (0.08-0.3 Hz) and B0H (0.3-1.0 Hz), to study the importance of the FWL components up to 0.3 Hz, separately from components above 0.3 Hz. Furthermore, we identified two new promising frequency bands, B0L’ (0.125-0.575 Hz) and B0L” (0.225-0.475 Hz), both believed to be related to the known FWL. The Peak Amplitude of the normalized power spectrum (*PA*), extracted from signal S3 in band B0L’, exhibited the lowest *p*-values to separate between later premature and term records among all of the studied features [81]. The Median Frequency (*MF*) and Peak Frequency (*PF*) of the power spectrum exhibited the lowest *p*-values when extracted from the newly defined band B0L. Furthermore, in [81] we also found high separability between later premature and term records in the band B3 (3.5-5.0 Hz), reflective of the influence of the maternal heart [57], and in the band B0b (0.08-4.0 Hz) [59] with the broadest frequency range. For the extraction of features in this study, we used all of the frequency bands previously used in [81], except the band B0H (0.3-1.0 Hz), which exhibited the poorest separability power overall. Therefore, in the present study, the following 10 bands were used for the extraction of features:

- non-overlapping bands B0 (0.08-1.0 Hz) [57], mainly reflective of the uterine physiological mechanisms, and B1 (1.0-2.2 Hz), B2 (2.2-3.5Hz), and B3 (3.5-5.0 Hz) [57], reflective of the influence of the maternal heart on the uterus in terms of the maternal heart rate, its 2nd, and 3rd harmonic, respectively,
- bands Bb (0.3-4.0 Hz) and B0b (0.08-4.0 Hz) with broad frequency ranges, expected to encompass physiological mechanisms of many etiologies [59],
- previously defined band B0L (0.08-0.3 Hz) for studying the importance of FWL components below 0.3 Hz, separately from components above 0.3 Hz [81],
- previously identified bands B0L’ (0.125-0.575 Hz) and B0L” (0.225-0.475 Hz), reflective of the FWL, as well as band B0H’ (0.575-1.0 Hz), mostly reflective of the FWH up to 1.0 Hz [81].

#### Features

Features, or parameters, are used to quantitatively gauge the selected characteristic of the non-stationary and time-varying EHG signals, which reflect the underlying non-linear physiological mechanisms [51, 59]. In clinical practice, it is highly desirable to use meaningful features that provide direct insight into the spectral and non-linear properties of the signals in the selected frequency bands related to known physiological mechanisms. In particular, spectral features are used to directly assess the frequency content, and entropy features to estimate the predictability, or regularity, of the EHG signals in the selected frequency bands.

Despite the increasing tendency of contemporary studies to use complex features, in this study, we limited our selection to simple, easily derivable, and understandable features. Although the use of more complex features might result in improved classification performance, it would significantly reduce overall explainability. In particular, we extracted three spectral parameters and one entropy parameter from the EHG signals in the selected frequency bands. The spectral parameters include the Median Frequency (*MF*) and the Peak Frequency (*PF*) of the power spectrum [59], and the Peak Amplitude of the normalized power spectrum (*PA*) [57]. These features were previously shown to exhibit significant powers to separate between premature and term records in a variety of frequency bands [81]. Furthermore, we calculated the Sample Entropy (*SE*) [60], as it is one of the most widely used entropy parameters for the analysis of EHG records [51]. Besides, it is also one of the most studied entropy measures in terms of interpretability in the context of biological signals [89, 90].

The Peak Frequency of the power spectrum [59], *PF*, in the selected frequency band was calculated from the power spectrum *P*(*k*) of the filtered signal according to:

$$\begin{array}{c} PF=\frac{f_{\text{S}}}{M}\text{arg}({\text{max}}_{k=k_{\text{L}}}^{k=k_{\text{U}}}P\left(k\right))\;, \end{array}$$ (1)

where *f*_S_ is the sampling frequency, *M* is number of components of *P*(*k*), and *k*_L_ and *k*_U_ are the indexes of *P*(*k*) at the lower and upper bounds of the power spectrum within which the peak frequency is searched for, respectively.

The Median Frequency of the power spectrum [59], *MF*, in the selected frequency band was calculated according to:

$$\begin{array}{c} MF=k_{\text{j}}\frac{f_{\text{S}}}{M}\;,\;\;\sum_{k=k_{\text{L}}}^{k=k_{\text{j}}}P\left(k\right)\;\approx \sum_{k=k_{\text{j}}+1}^{k=k_{\text{U}}}P\left(k\right)\;. \end{array}$$ (2)

To calculate the Peak Amplitude of the normalized power spectrum [57, 81], *PA*, we first calculated the reference peak amplitude, *PA*_REF_, of the power spectrum of the filtered signal in the reference frequency band B0 (0.08-1.0 Hz), *P*_R_(*k*), according to:

$$\begin{array}{c} PA_{\text{REF}}={\text{max}}_{k=k_{\text{RL}}}^{k=k_{\text{RU}}}P_{\text{R}}\left(k\right)\;, \end{array}$$ (3)

where *k*_RL_ and *k*_RU_ are the indexes of *P*_R_(*k*) at the lower and upper bounds of the reference frequency band. Then, using the power spectrum of the filtered signal in the selected frequency band, we calculated *PA* according to:

$$\begin{array}{c} PA={\text{max}}_{k=k_{\text{L}}}^{k=k_{\text{U}}}\frac{P(k)}{PA_{\text{REF}}}\;, \end{array}$$ (4)

where *k*_L_ and *k*_U_ are the indexes of *P*(*k*) at the lower and upper bounds of the selected frequency band.

Less regular and more predictable signals, or time series, exhibit lower entropy, and several studies have shown that Sample Entropy [60], *SE*, of EHG signals decreases as pregnancy progresses (complexity of the signal decreases as the signal becomes more regular as labor approaches) [59, 63]. To calculate *SE*, first let *x*(*n*), 0 < *n* ≤ *N*, denote the time series with *N* samples. The template vectors *X*_*m*_(*i*) with a length of *m*, or embedding dimension, where *m* < *N*, are constructed from *x*(*n*) according to:

$$\begin{array}{c} X_{m}\left(i\right)=\left\{x\left(i\right),x\left(i+1\right)\;,\ldots ,\;x\left(i+m-1\right)\right\}\;, \end{array}$$ (5)

where 0 < *i* ≤ *N* − *m* + 1. The distance function between a pair of vectors *X*_*m*_(*i*) and *X*_*m*_(*j*), is defined as the Chebyshev distance between the vector pairs according to:

$$\begin{array}{c} d\left(m,i,j\right)=D(X_{m}\left(i\right),X_{m}\left(j\right))=\;max\{|X_{m}\left(i\right)-X_{m}\left(j\right)\left|\right\}\;, \end{array}$$ (6)

where 0 < *k* ≤ *m*. Two vectors are considered a match if *d*(*m*, *i*, *j*) ≤ *r*, where *r* denotes the distance threshold, or tolerance. Let *c*_*m*_ denote the number of matches between vectors *X*_*m*_(*i*) and *X*_*m*_(*j*), where *i*, *j* ∈ [1, *N* − *m* + 1] and *i* ≠ *j*. Then, for a given *m* and *r*, *SE*(*m*, *r*) is defined as:

$$\begin{array}{c} SE\left(m,r\right)=\{\begin{array}{cc} -ln(c_{m+1}/c_{m}) & ;\;\;c_{m+1}\neq 0\wedge c_{m}\neq 0\\ -ln(\frac{N-m}{N-m-1}) & ;\;\;\text{otherwise} \end{array}\;. \end{array}$$ (7)

The tolerance value *r* is usually specified as a fraction of the standard deviation of the input time series. In the present work, we used the most commonly used embedding dimension and tolerance value for assessing the regularity of EHG signals, *m* = 3 and *r* = 0.15, respectively [57–59].

### Feature selection and classification

The use of the traditional feature engineering approach commonly employs a complex, high-dimensional feature space, which requires prior selection, or reduction of the number, of features to achieve acceptable classifier generalization. Even so, many methods rely on using tens or even hundredths of features, as well as “black-box” approaches based on deep learning, which may severely diminish explainability. In contrast, in this study we used at most two easily derivable and understandable features for classification of premature and term records, thereby reducing the input feature space to at most two dimensions. The most promising features were identified using the selected feature selection strategy, using multiple standard classifiers and feature sets. For feature selection and classifier performance evaluation, we initially used only later premature (group PL) and later term spontaneous records (group TL). Next, to assess the impact of common non-spontaneous term delivery modes, we repeated the feature selection and classifier performance evaluation, this time using later premature (group PL) and later term spontaneous, induced, cesarean, and induced-cesarean records (groups TL, IL, CL, and ICL). To address the premature and term class imbalance, we strictly adhered to the proposed Partition-Synthesis evaluation technique [70]. Therefore, the minority class was over-sampled in each repetition of feature selection and classification only after partitioning the data into training and test sets.

#### Selected classifiers

For feature selection and classification, we selected several traditional classifiers that are most widely used in the area of automated prediction of premature birth. In particular, the classifiers used in this study include the widely used Linear Discriminant Analysis (LDA) [72, 91–93], Quadratic Discriminant Analysis (QDA) [57, 73, 76, 91–93], Naive Bayes [75, 94], and Support Vector Machine [66, 67, 77, 92, 93, 95] with third order polynomial kernel (SVM Poly3) and the Radial Basis Function kernel (SVM RBF). All of the models were implemented in Matlab (R2023b) using its native implementation of the above classifiers. No explicit hyper-parameter optimization was performed, and always default hyper-parameters and options were used, except for the following: i) For LDA and QDA, we used the pseudo-linear and pseudo-quadratic discriminants, respectively, instead of the standard linear and quadratic discriminants in order to assure classification without failure (i.e. when the inverse of the covariance matrix can not be calculated, the Moore-Penrose pseudoinverse is used); ii) For Naive Bayes, we used the Normal (Gaussian) data distribution to model the data and the empirical class distribution for the prior class probability distribution; and iii) For SVM Poly3 and SVM RBF, we used the used the Sequential Minimal Optimization (SMO) [96] solver instead of the default optimization routine to improve computational efficiency.

In our preliminary investigation of classification performance, we also tested the *k*-Nearest Neighbours (KNN) [68, 91–93] (*k* = 3) and Decision Tree (DT) [66, 68, 92, 93] (without tree depth limit and without pruning) classifiers. Among the classifiers used, KNN and DT performed the worst, and the related results are not included in this paper.

#### Identification of the most promising features

The full feature set includes *PF*, *MF*, *PA*, and *SE*, extracted from 10 frequency bands for signals S2 and S3. Note however, *PA* was not extracted from signals S2 and S3 in bands B0 and B0b, since when extracted from these bands the feature values equal 1.0 for all EHG records. Therefore, the full feature set includes 76 (80—4) features. The resulting high-dimensional feature space is complex, and using all of the features for classification may negatively impact the classification performance due to overfitting to the training data (resulting in poor generalization), and due to possible correlation between the features (redundant information). To decrease the number of features, simplify the feature space, reduce model complexity, mitigate the potential for overfitting, and improve classification performance, researchers employ various feature selection techniques [97].

Feature selection based on the Sequential Forward Selection (SFS) method [84] is a simple wrapper feature selection search strategy. In each step, the available feature that improves the desired classification performance metric the most (selection criterion) is sequentially added to the initially empty feature subset, either until a stopping criterion is met or until all of the features have been selected. The stopping criterion is based on the minimum of the average misclassification error (MCE), i.e. the number of misclassified samples divided by the total number of samples in the training data set. However, multiple runs of the SFS yield varying feature subsets. To avoid this inherent instability in the SFS method, the selection of features is stabilized with multiple runs of the SFS [85]. The occurrences of features in the yielded subsets are aggregated across all runs of the SFS. The resulting aggregated frequency histogram of feature occurrences shows the number of times that each feature was present in all of the yielded feature subsets. The final feature subset is constructed by using only the features having the highest numbers of occurrences in the aggregated frequency histogram, whereby the number of features in the final subset is determined by the minimum of the MCE function, averaged across all runs of the SFS.

The features were initially sorted according to the *p*-values to separate between premature and term records of the used dataset. For each (*i*-th) repetition of the SFS, we used the stratified hold-out strategy to divide the dataset into the training set *TR*_*i*_ (80% of the dataset) and the test set *TE*_*i*_ (20% of the dataset). The test sets were not used for feature selection, and remained reserved for the final classification performance evaluation with *N* repetitions, using the identified most promising features. The average MCE of the stratified 5-fold cross-validation, using only the training set (*TR*_*i*_) in each SFS repetition, was used to select the next best feature to be added to the feature subset until no more features remained to be selected. Inside the cross-validation loop for calculating the MCE in each repetition of the SFS, we used the Synthetic Minority Over-sampling technique (SMOTE) [98] to balance the premature and term classes. This was done only after splitting, or partitioning, the data into the training-validation subsets, according to the Partition-Synthesis evaluation technique [70]. The subset of selected features in each run of the SFS comprises the selected *K*_min_ features that yielded the minimum of the MCE function. Then, the aggregated frequency histogram of feature occurrences *H* was updated based on the subset of selected features in each SFS repetition. The number of final selected features was defined by the minimum of the average MCE function across all repetitions of the SFS.

Algorithm 1 describes the used feature selection procedure in more detail, where *X* is the input feature set with *K* features, *N* is the number of repetitions of the SFS, Dataset is the input set of premature and term records, function MCE returns the average cross-validation misclassification error using the specified subset of data and features, and function Index returns the position of the selected feature in the histogram. The feature selection procedure returns the aggregated frequency histogram of feature occurrences *H*, and the final selected feature subset *Y* with *m* features.

**Algorithm 1** Pseudo code for feature selection using SFS, stabilized with the frequency-based aggregation of the selected features technique

**Input**: *X* = {*x*_1_, *x*_2_, …, *x*_*K*_}, *N* = 200, and Dataset

**Output**: histogram *H*, number of final selected features *m*,

    and final selected feature subset *Y*

 *H*_*k*_ = *M*_*k*_ = 0, *k* ∈ [1, *K*] // Initialize values

 *Y* = Sort_*p*_(*X*) // Sort *X* according to *p*-values

 **for** *i* = 1 to *N* **do**

  [*TR*_*i*_, *TE*_*i*_] = Partition(Dataset) // Holdout 80%/20%

  *Z* = ∅ // Start with empty feature subset

  **for** *k* = 1 to *K* **do** // Sequential forward selection

$z_{k}={\text{arg}\;\text{min}}_{\left(y_{k}\in Y\right)\wedge \left(y_{k}\notin Z\right)}\left(\text{MCE}\left(T\;R_{i},Z\cup y_{k}\right)\right)$

   *M*_*k*_ = *M*_*k*_ + MCE(*TR*_*i*_, *Z* ∪ *z*_*k*_)

   *Z* = *Z* ∪ *z*_*k*_ // Add selected feature to subset

  **end for**

  // Find subset yielding the lowest MCE

  *K*_min_ = arg min_*k*∈[1,*K*]_(MCE(*TR*_*i*_, {*z*_1_, …, *z*_*k*_}))

  // Update histogram values

  **for** *k* = 1 to *K*_min_ **do**

$H_{\text{Index}(z_{k})}=H_{\text{Index}(z_{k})}+1$

  **end for**

 **end for**

 // Get number of final selected features

 *m* = arg min_*k*∈[1,*K*]_(*M*_*k*_/*N*)

 // Construct final subset based on histogram peaks

 *Y* = ∅

 **for** *k* = 1 to *m* **do**

$f={\text{arg}\;\text{max}}_{\left(f\in \left[1,K\right]\right)\wedge \left(x_{f}\notin Y\right)}\left(H_{f}\right)$

  *Y* = *Y* ∪ *x*_*f*_

 **end for**

To reduce bias towards a particular classifier used for calculating the average MCE function, and to avoid emphasizing features extracted from less promising broad frequency bands, the whole feature selection procedure was performed multiple times, each time using one of the five selected classifiers (LDA, QDA, Naive Bayes, SVM Poly3, and SVM RBF) and one of the following four feature sets:

- Feature set 1: The four features, *PF*, *MF*, *PA*, and *SE*, extracted from signals S2 and S3 in all 10 of the selected frequency bands, except *PA* for S2 and S3 in bands B0 and B0b (in total 4 ⋅ 2 ⋅ 10 − 4 = 76 features);
- Feature set 2: All features from feature set 1, except those extracted from the least promising bands B0H and Bb (features extracted from these two bands yielded the least significant *p*-values to separate between premature and term records according to [81]) (4 ⋅ 2 ⋅ 8 − 4 = 60 features);
- Feature set 3: The four features, *PF*, *MF*, *PA*, and *SE*, extracted from signals S2 and S3, but only in bands B0L, B0L’, B3 (most promising bands according to [81]), and the broadest band B0b, except *PA* for S2 and S3 in band B0b (4 ⋅ 2 ⋅ 4 − 2 = 30 features);
- Feature set 4: All features from feature set 3, except those extracted from band B0b (4 ⋅ 2 ⋅ 3 = 24 features).

**Fig 1:**
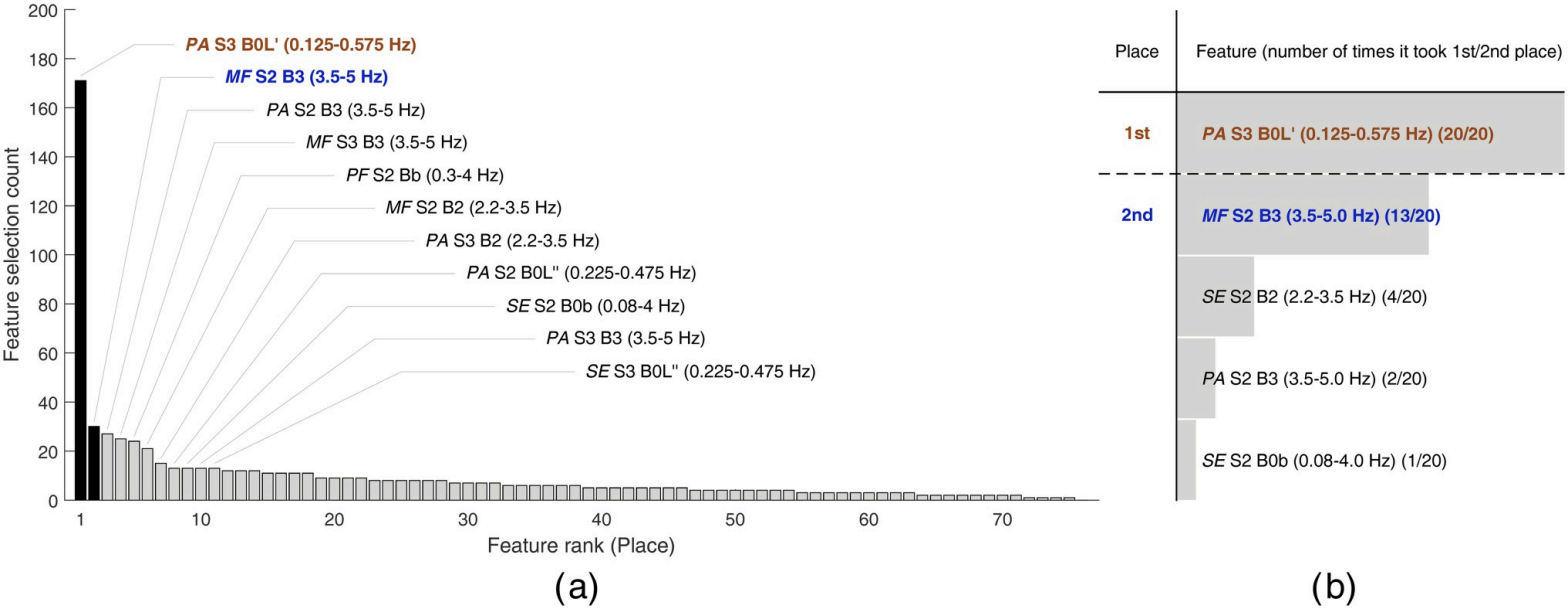
The results of feature selection using later premature (group PL) and later term spontaneous (group TL) records. The left histogram (a) shows the counts of feature occurrences and the optimal set of features yielded by the feature selection procedure if using feature set 1 and LDA classifier. Features are ranked according to the feature occurrence count. The features from the optimal set are annotated, and the bars for the top two features (i.e. the features taking the first and second place) are shown in black. The right histogram (b) shows the total number of times that a feature took first place (above the dashed separator line) and second place (below the dashed separator line) out of 20 runs (i.e. using five classifiers and four feature sets) of the feature selection procedure.

**Fig 2:**
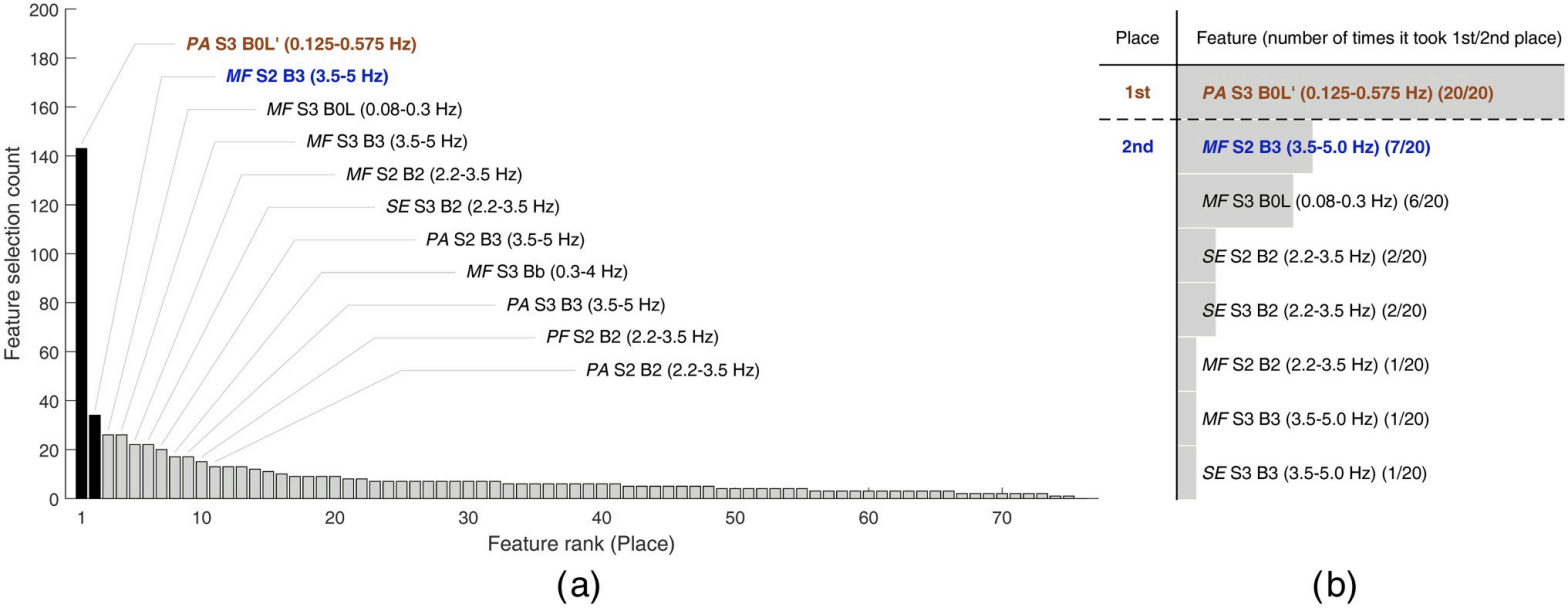
The results of feature selection using later premature (group PL) and later term spontaneous, induced, cesarean, and induced-cesarean (groups TL, IL, CL, and ICL) records. The left histogram (a) shows the counts of feature occurrences and the optimal set of features yielded by the feature selection procedure if using feature set 1 and LDA classifier. Features are ranked according to the feature occurrence count. The features from the optimal set are annotated, and the bars for the top two features (i.e. the features taking the first and second place) are shown in black. The right histogram (b) shows the total number of times that a feature took first place (above the dashed separator line) and second place (below the dashed separator line) out of 20 runs (i.e. using five classifiers and four feature sets) of the feature selection procedure.

In total, the whole feature selection procedure was performed 20 times (for five classifiers * four feature sets), each time yielding one histogram (examples of these histograms are shown in Figs 1(a) and 2(a) in the Results section) for each distinct classifier and feature set combination. Although it is expected that each run of the feature selection procedure returns the final subset with more than two features, in this study we intentionally chose to perform the classification between premature and term record using only one, or at most two, of the most promising features. If using a single feature, or a single biomarker, for classification, the decision boundary between premature and term classes is simply represented with a single value, i.e. a threshold. In the case of using two features, the two-dimensional feature space is easily comprehensible, and the decision boundary can be very effectively visualized. Therefore, only the highest two peaks of each histogram of feature occurrences were noted (indicating the two of the most selected features, i.e. the features taking the first and the second place, for each individual classifier and feature set combination). The number of times that a feature took first place (feature A) or second place (feature B) was then counted for all combinations of classifiers and feature sets (five classifiers * four feature sets). This yielded a final histogram that shows how many times the feature took the first or second place, out of 20 runs of the feature selection procedure. These histograms are shown to summarize our results in Figs 1(b) and 2(b) in the Results section. Finally, the two features that most often took the first or second place in the final histogram were identified as the two most promising features (feature A and feature B).

#### Evaluation technique

After two of the most promising features (A, B) were identified, we evaluated the classification performance of the selected classifiers when using either one of the features, and when using both features (A and B). The evaluation of classification performance was repeated *N* = 200 times (i.e. the same as the number of repetitions of the SFS procedure). For training and evaluating the classifier, we used the existing data partitions (training and test sets) from the corresponding repetitions of the SFS feature selection procedure. Notably, for each repetition of the evaluation, the test set represents the data that was not used in the prior feature selection and the training steps. Moreover, the data was already split into the training and test sets beforehand. Hence, subsequent over-sampling of the minority class, using SMOTE [98], was performed after data partitioning in accordance with the Partition-Synthesis evaluation technique [70].

For each classifier and repetition of performance evaluation, we calculated the Sensitivity (*Se*), Specificity (*Sp*), Positive Predictive Value (*PPV*), Negative Predictive Value (*NPV*), Classification Accuracy (*CA*), and the Area Under the ROC Curve (*AUC*) [99], using the counts of correctly predicted premature records (*TP*), incorrectly predicted premature records (*FN*), correctly predicted term records (*TN*), and incorrectly predicted term records (*FP*), according to:

$$\begin{array}{c} Se=\frac{TP}{TP+FN}\;, \end{array}$$ (8)

$$\begin{array}{c} Sp=\frac{TN}{TN+FP}\;, \end{array}$$ (9)

$$\begin{array}{c} PPV=\frac{TP}{TP+FP}\;, \end{array}$$ (10)

$$\begin{array}{c} NPV=\frac{TN}{TN+FN}\;, \end{array}$$ (11)

$$\begin{array}{c} CA=\frac{TP+TN}{TP+FP+TN+FN}\;, \end{array}$$ (12)

$$\begin{array}{c} AUC=\int_{0}^{1}Se\left(1-Sp\right)\;d\left(1-Sp\right)\;. \end{array}$$ (13)

Then, the average values of *Se*, *Sp*, *PPV*, *NPV*, *CA*, and *AUC* for each of the used classifiers were calculated across all repetitions using the corresponding test sets. Additionally, we compared these results to those obtained on the corresponding training sets in order to assess whether the classification models generalize well to unseen data. Furthermore, we also calculated the average ROC curves with a simple non-parametric method by averaging the sensitivity at each different specificity value [99, 100].

#### Visualization of average decision boundaries

**Fig 3:**
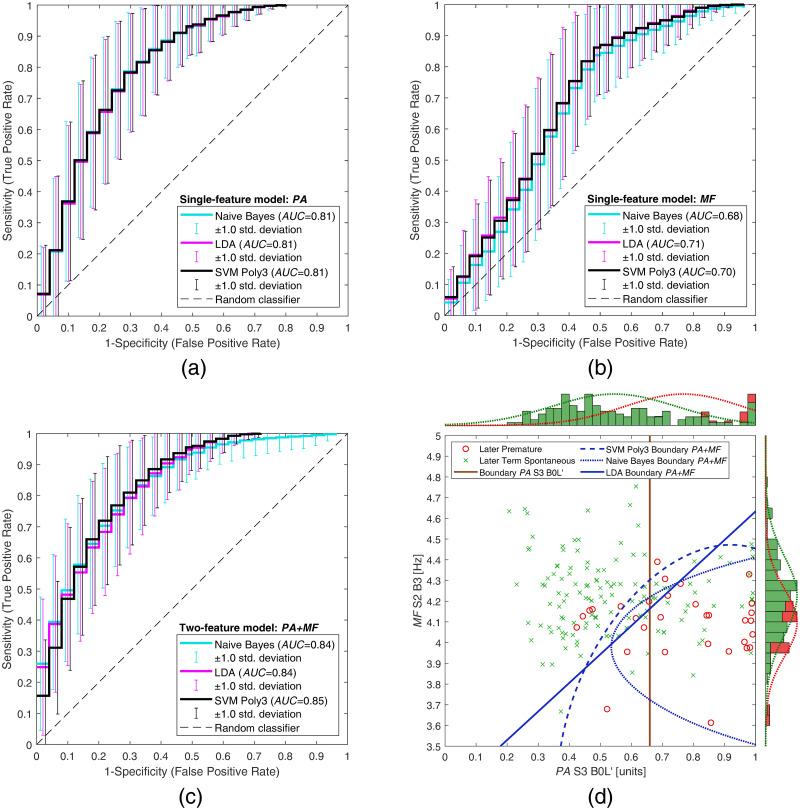
Average ROC curves and decision boundaries for classification between later premature records (group PL) and later term spontaneous records (group TL). The average ROC curves are shown for classification (a) using only feature *PA*, (b) using only feature *MF*, and (c) using two features *PA*+*MF*, for the Naive Bayes (shown in cyan), LDA (shown in magenta), and SVM Poly3 (shown in black) classifiers. The error bars for each ROC curve show the standard deviations at different false positive rates. In the scatter plot (d), the average classification decision boundaries are shown for the single-feature model (*PA*), averaged across all classifiers (vertical brown boundary), and for two-feature models (*PA*+*MF*) using Naive Bayes (dotted blue boundary), LDA (solid blue boundary), and SVM Poly3 (dashed blue boundary) classifiers. The histograms on the top and right sides of the scatter plot show the normalized distribution of the feature values for premature (in red) and term records (in green). The red and green dotted lines on the histograms show the fit to the normal distribution according to the feature values for premature and term records, respectively.

**Fig 4:**
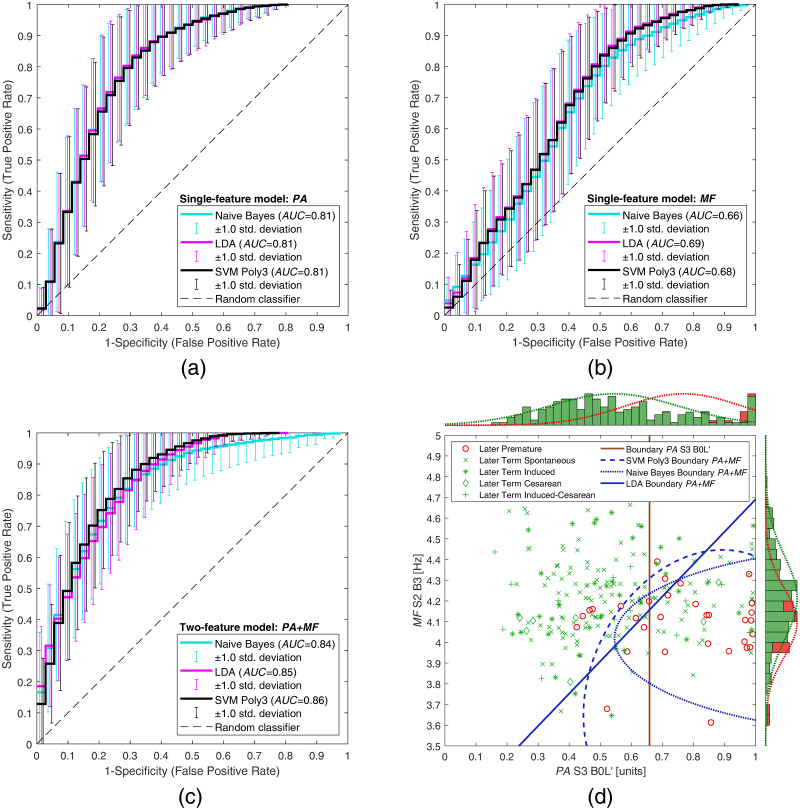
Average ROC curves and decision boundaries for classification between later premature records (group PL) and all later term records (groups TL, IL, CL, ICL). The average ROC curves are shown for classification (a) using only feature *PA*, (b) using only feature *MF*, and (c) using two features *PA*+*MF*, for the Naive Bayes (shown in cyan), LDA (shown in magenta), and SVM Poly3 (shown in black) classifiers. In the scatter plot (d), the average classification decision boundaries are shown for the single-feature model (*PA*), averaged across all classifiers (vertical brown boundary), and for two-feature models (*PA*+*MF*) using Naive Bayes (dotted blue boundary), LDA (solid blue boundary), and SVM Poly3 (dashed blue boundary) classifiers. The histograms on the top and right sides of the scatter plot show the normalized distribution of the feature values for premature (in red) and term records (in green). The red and green dotted lines on the histograms show the fit to the normal distribution according to the feature values for premature and term records, respectively.

In case of using a single feature for binary classification between premature and term records, the task of all of the selected classifiers is reduced to simple thresholding of the feature value. Hence, all of the trained classifiers may be modeled with a single parameter, or threshold. Nevertheless, even in the case of a single feature, different algorithms for training the selected classifiers may produce varying thresholds, resulting in varying sensitivity-specificity trade-offs. Furthermore, the evaluation technique with multiple repetitions (*N* = 200), each time using different subsets of the data for training, resulted in different threshold values. Therefore, for the case of using a single feature, we calculated the average threshold across all repetitions and classifiers. This resulted in the average threshold value for the single feature, regardless of classifier. This single threshold reflects the learned decision boundary between premature and term records in a one-dimensional feature space. It can also be visualized in the two-dimensional feature space with a single straight line, perpendicular to the axis of the thresholded feature (these boundaries are shown in Figs 3(d) and 4(d) in the Results section).

If using two features, the decision boundary curve depends on the classifier. To visualize the average decision boundary of the binary classifiers between premature and term records, we constructed a grid of feature values in the two dimensional feature space, and performed the classification using the feature values across the grid. This yielded a matrix of classifier scores, indicating the likelihood that the sample at the individual position in the grid comes from the premature class. These scores were then averaged across all repetitions, and a single contour, or isoline (at the level of the 50% likelihood that the sample belongs to the premature class), was drawn for the best performing classifiers on the scatter plot of feature values for all records of the used datasets.

## Results

### The most promising selected features

Using later premature (group PL) and later term spontaneous (group TL) records of the TPEHG DB and TPEHGT DS, we first identified the most promising features based on the results of the SFS method, stabilized with the frequency-based aggregation of the selected features technique, with the specified number of repetitions (*N* = 200), using various classifiers and feature sets. The feature selection procedure always returned the final feature subset with more than two features, regardless of classifier and feature set. The upper half of Table 4 shows the optimal number of features returned by the feature selection procedure and the achieved average Classification Accuracy, *CA*, on the validation sets, when performing feature selection and classification between later premature (group PL) and later term spontaneous (group TL) records, using various classifiers and feature sets. The highest *CA* (83.4% ± 3.1%) was achieved with the optimal set comprising nine features returned by the feature selection procedure using the SVM Poly3 classifier and feature set 1. The average optimal number of returned features, across all classifiers, was 13.0 ± 8.0, 12.2 ± 6.6, 7.4 ± 3.2, and 6.8 ± 2.0, when using feature set 1, 2, 3, and 4, respectively. Even so, to identify the two most promising features, we always used only the two highest peaks (features taking first and second place) in the histogram of feature occurrences.

**Table 4:** The optimal number of features, and the achieved Classification Accuracy (*CA*), when performing the feature selection, and classification on the validation sets, with the specified number of repetitions (*N* = 200), using various classifiers and feature sets.

Classifier	Feature set 1		Feature set 2		Feature set 3		Feature set 4	
	Features	*CA* [%]	Features	*CA* [%]	Features	*CA* [%]	Features	*CA* [%]
Using later premature records (group PL) and later term spontaneous records (group TL)								
SVM Poly3	9	**83.4** ± **3.1**	11	83.0 ± 3.2	7	81.2 ± 2.9	7	80.3 ± 2.8
SVM RBF	27	80.3 ± 3.0	23	79.6 ± 2.8	13	78.2 ± 2.7	10	77.4 ± 2.7
Naive Bayes	11	82.1 ± 2.9	8	81.6 ± 2.7	6	78.9 ± 2.7	7	78.2 ± 2.8
LDA	11	80.7 ± 3.2	13	80.1 ± 3.3	6	78.3 ± 2.7	5	78.1 ± 2.6
QDA	7	81.4 ± 3.1	6	81.0 ± 3.0	5	78.8 ± 2.5	5	78.2 ± 2.7
Using later premature records (group PL) and all later term records (groups TL, IL, CL, and ICL)								
SVM Poly3	10	**85.0** ± **3.3**	9	84.4 ± 3.1	7	82.7 ± 2.9	7	82.3 ± 2.8
SVM RBF	21	81.1 ± 3.0	19	82.5 ± 3.1	10	81.0 ± 2.7	9	80.3 ± 2.8
Naive Bayes	14	83.4 ± 2.6	10	82.8 ± 2.7	6	80.0 ± 2.7	5	79.6 ± 2.8
LDA	11	82.7 ± 3.1	12	82.5 ± 3.0	7	80.0 ± 3.1	7	79.8 ± 3.2
QDA	6	82.4 ± 3.1	5	81.4 ± 3.1	4	79.3 ± 3.2	4	78.8 ± 3.4

The upper half of table shows the results when feature selection and classification were performed using later premature records (group PL) and later term spontaneous records (group TL) of the TPEHG DB and TPEHGT DS. The lower half of table shows the results when feature selection and classification were performed using later premature records (group PL) and later term spontaneous, induced, cesarean, and induced-cesarean records (groups TL, IL, CL, and ICL) of the TPEHG DB, TPEHGT DS, and ICEHG DS. Models: SVM Poly3—Support Vector Machine with third order polynomial kernel, SVM RBF—Support Vector Machine with Radial Basis Function kernel, LDA—Linear Discriminant Analysis, QDA—Quadratic Discriminant Analysis.

The left histogram in Fig 1(a) shows the counts of feature occurrences returned by the feature selection procedure if using feature set 1 and the LDA classifier with records of the TPEHG DB and TPEHGT DS. In particular, the feature selection procedure using this feature set and LDA classifier yielded an optimal set with 11 features (according to upper part of Table 4) which are annotated. Note the two highest peaks of the histogram, i.e. the first two black bars highlighting the two features having the highest feature occurrence count. In this particular case, out of *N* = 200 repetitions, feature *PA* of signal S3 in the frequency band B0L’ (0.125-0.575 Hz), succinctly *PA* S3 B0L’, ended in the selected feature subset 171 times (taking first place among all of the features when using this classifier and feature set). Moreover, feature *MF* of signal S2 in band B3 (3.5-5.0 Hz), succinctly *MF* S2 B3, ended in the feature subset 30 times (taking second place). The feature selection procedure was repeated for all five classifiers and four feature sets, yielding 20 such histograms. The right histogram in Fig 1(b) shows the number of times features took first place (above the dashed separator line) and the number of times features took second place (below the dashed separator line) out of 20 runs of the feature selection procedure (using five classifiers and four feature sets). *PA* S3 B0L’ was always the most selected feature, regardless of classifier and feature set, hence it took first place in 20 out of 20 runs of the feature selection procedure. This is why the histogram above the dashed separator line in Fig 1(b) shows only one bar. However, the second place is shared by four features, as shown in the histogram below the dashed separator line in Fig 1(b). This means that for some classifiers and feature sets, the second most selected feature was either *MF* S2 B3, *SE* S2 B2, *PA* S2 B3, or *SE* S2 B0b. Nevertheless, *MF* S2 B3 took the second place among the selected features in nine out of 20 runs, which is above the counts for other features sharing the second place, as shown in Fig 1(b). Based on these results, *PA* S3 B0L’ and *MF* S2 B3 were identified as two of the most promising features for classification between later premature (group PL) and later term spontaneous (group TL) records.

The feature selection was also performed for classification between later premature (group PL) and later term spontaneous, induced, cesarean, and induced-cesarean (groups TL, IL, CL, and ICL) records of the TPEHG DB, TPEHGT DS, and ICEHG DS. For this case, the optimal number of features returned by the feature selection procedure and the achieved average *CA* on the validation sets are shown in the lower half of Table 4, using various classifiers and feature sets. The highest *CA* (85.0% ± 3.3%) was achieved with the optimal set comprising 10 features returned by the feature selection procedure using the SVM Poly3 classifier and feature set 1. The average optimal number of returned features, across all classifiers, was 12.4 ± 5.6, 11.0 ± 5.1, 6.8 ± 2.2, and 6.4 ± 1.9, when using feature set 1, 2, 3, and 4, respectively. Again, to subsequently identify the two most promising features, we always used only the features taking first and second place. Fig 2(a) shows the resulting histogram of feature occurrences returned by the feature selection procedure if using feature set 1 and LDA classifier which yielded an optimal set with 11 features (according to lower half of Table 4). Similarly to the results in Fig 1(a), *PA* S3 B0L’ was the most selected feature, followed by *MF* S2 B3. Next, Fig 2(b) shows the histogram with the number of times that features took the first place (shown in the histogram above the dashed separator line) and second place (shown in the histogram below the dashed separator line) out of 20 runs of the feature selection procedure. Again, feature *PA* S3 B0L’ took first place in 20 out of 20 runs of the feature selection procedure, as it was always the most selected feature, regardless of the used classifier and feature set. The second place is shared by seven features, and of those *MF* S2 B3 most often took second place (in seven out of 20 runs). Interestingly, regardless of the dataset used (either only using records from groups PL and TL, or using records from groups PL, TL, IL, CL, and ICL), the same two features, *PA* S3 B0L’ and *MF* S2 B3, were identified as most promising.

### Classification performance

Table 5 summarizes the classification performance results (on the *N* = 200 leftover test sets that were not used for feature selection) for classification between later premature (group PL) and later term spontaneous (group TL) records, using one or two of the previously identified most promising features, (A) *PA* S3 B0L’ and (B) *MF* S2 B3, a variety of standard classifiers. If using only feature (A), *PA*, the *AUC* score is 0.81 ± 0.08, regardless of the used classifier. The Sensitivity (*Se*), Specificity (*Sp*), Positive Predictive Value (*PPV*), Negative Predictive Value (*NPV*), and Classification Accuracy (*CA*) slightly vary depending on the classifier, due to differences in the training algorithms. Using only feature (A), *PA*, the LDA classifier yielded the highest *CA* (75.1% ± 9.1%), while the Naive Bayes classifier yielded the next highest *CA* (74.6% ± 9.3%). If using only the second most promising feature (B), *MF*, the classification performance results are significantly worse. However, if using using both features (A and B), the *CA* and *AUC* are both slightly higher, compared to using a single feature. In particular, the best results were obtained using the SVM Poly3 classifier, yielding the highest *CA* (76.3% ± 7.8%) and *AUC* (0.85 ± 0.07). For two features (A and B), SVM Poly3 exhibited the highest *Se* (86.0% ± 18.6%) and *NPV* (82.6% ± 12.8%) among all classifiers, at the cost of lower *Sp* (66.6% ± 8.3%) and *PPV* (72.0% ± 6.2%), respectively, while other classifiers, particularly LDA and Naive Bayes, yielded more balanced *Se* and *Sp* (and more balanced *PPV* and *NPV*), with only slightly reduced *AUC* (0.84 ± 0.08). Furthermore, the average *AUC* and *CA* obtained on the testings sets (Table 5) were very close to those obtained on the training sets. In particular, using either one (A or B) or both features (A and B), the average *CA* and *AUC* using the testing sets were never lower by more than 1.8% and 0.02, respectively, compared to using the training sets, regardless of feature set and classifier, suggesting that all of these models generalize well to unseen data.

**Table 5:** Classification performance results for the top two features (*PA* and *MF*), and of their combination, when classifying between later premature records (group PL) and later term spontaneous records (group TL) of the TPEHG DB and TPEHGT DS.

Feature set	Classifier	*Se* [%]	*PPV* [%]	*Sp* [%]	*NPV* [%]	*CA* [%]	*AUC*
Single feature: (A) *PA* S3 B0L’ (0.125-0.575 Hz)	SVM Poly3	68.9 ± 19.5	75.0 ± 8.2	77.0 ± 8.1	71.2 ± 13.1	72.9 ± 9.8	0.81 ± 0.08
	SVM RBF	75.2 ± 17.7	73.3 ± 7.5	72.6 ± 8.9	74.5 ± 13.0	73.9 ± 9.2	0.81 ± 0.08
	Naive Bayes	75.0 ± 17.8	74.4 ± 7.3	74.3 ± 8.0	74.8 ± 12.9	74.6 ± 9.3	0.81 ± 0.08
	LDA	74.4 ± 17.4	75.4 ± 7.7	75.8 ± 7.9	74.7 ± 12.9	**75.1** ± **9.1**	**0.81** ± **0.08**
	QDA	74.7 ± 18.1	74.4 ± 7.3	74.3 ± 7.8	74.6 ± 12.9	74.5 ± 9.7	0.81 ± 0.08
Single feature: (B) *MF* S2 B3 (3.5-5.0 Hz)	SVM Poly3	79.6 ± 18.6	64.9 ± 7.0	56.9 ± 9.2	73.6 ± 16.3	68.3 ± 9.2	0.70 ± 0.10
	SVM RBF	85.0 ± 14.2	64.5 ± 5.4	53.2 ± 9.0	78.0 ± 15.3	69.1 ± 7.5	0.71 ± 0.10
	Naive Bayes	79.4 ± 15.6	63.8 ± 6.2	55.0 ± 9.2	72.7 ± 14.5	67.2 ± 8.9	0.68 ± 0.09
	LDA	70.4 ± 19.0	65.6 ± 8.5	63.1 ± 8.5	68.1 ± 15.4	66.8 ± 10.1	0.71 ± 0.10
	QDA	79.5 ± 15.2	62.4 ± 6.2	55.2 ± 9.1	68.4 ± 15.2	67.4 ± 8.9	0.68 ± 0.10
Two features (A and B): (A) *PA* S3 B0L’ (0.125-0.575 Hz) (B) *MF* S2 B3 (3.5-5.0 Hz)	SVM Poly3	86.0 ± 13.8	72.0 ± 6.2	66.6 ± 8.3	82.6 ± 12.8	**76.3** ± **7.8**	**0.85** ± **0.07**
	SVM RBF	76.6 ± 18.1	74.2 ± 7.9	73.3 ± 8.4	75.8 ± 13.5	75.0 ± 9.5	0.85 ± 0.08
	Naive Bayes	76.2 ± 17.7	74.7 ± 7.6	74.2 ± 8.0	75.7 ± 13.1	75.2 ± 8.9	0.84 ± 0.08
	LDA	74.1 ± 18.2	75.1 ± 7.5	75.4 ± 8.1	74.5 ± 12.7	74.8 ± 9.5	0.84 ± 0.08
	QDA	76.0 ± 18.0	74.8 ± 7.6	74.4 ± 7.9	75.6 ± 13.3	75.2 ± 9.1	0.83 ± 0.08

The table shows the means and standard deviations for Sensitivity (*Se*), Specificity (*Sp*), Positive Predictive Value (*PPV*), Negative Predictive Value (*NPV*), Classification Accuracy (*CA*), and Area Under the ROC Curve (*AUC*) with *N* = 200 repetitions. For the best single-feature set (A), the highest *CA* and associated *AUC* are in bold and shaded in gray, and the next highest *CA* and associated *AUC* are only shaded in gray. For the two-feature set (A and B), the highest *CA* and associated *AUC* are in bold and shaded in gray. SVM Poly3—Support Vector Machine with third order polynomial kernel, SVM RBF—Support Vector Machine with Radial Basis Function kernel, LDA—Linear Discriminant Analysis, QDA—Quadratic Discriminant Analysis.

Similarly, Table 6 summarizes the classification performance results (on the *N* = 200 leftover test sets that were not used for feature selection) for classification between later premature (group PL) and later term spontaneous, induced, cesarean, and induced-cesarean (groups TL, IL, CL, and ICL) records. These results are comparable to the performance results for classification between records from groups PL and TL only (shown in Table 5). If using only the most promising feature (A), *PA*, the Naive Bayes and the LDA classifiers performed the best, with *CA* (76.5% ± 8.9%) and (76.4 ± 9.2%), respectively, and yielding the same *AUC* (0.81 ± 0.08). In comparison, the results for the second most promising feature (B), *MF*, are significantly worse. Furthermore, if using both features (A and B), SVM Poly3 exhibited the highest classification performance, yielding the highest *CA* (78.0% ± 7.1%) and *AUC* 0.86 ± 0.07, with the highest *Se* (88.1% ± 12.3%) and *NPV* (85.1% ± 11.6) at the cost of lower *Sp* (67.8% ± 8.1%) and *PPV* (73.3% ± 6.0), respectively. Similarly, the average *CA* and *AUC* using the testing sets (Table 6) were never lower by more than 1.8% and 0.01, respectively, compared to using the training sets, regardless of feature set and classifier.

**Table 6:** Classification performance results for the top two features (*PA* and *MF*), and of their combination, when classifying between later premature records (group PL) and later term spontaneous, induced, cesarean, and induced-cesarean records (groups TL, IL, CL, and ICL) of the TPEHG DB, TPEHGT DS, and ICEHG DS.

Feature set	Classifier	*Se* [%]	*PPV* [%]	*Sp* [%]	*NPV* [%]	*CA* [%]	*AUC*
Single feature: (A) *PA* S3 B0L’ (0.125-0.575 Hz)	SVM Poly3	75.1 ± 19.1	74.7 ± 8.3	74.6 ± 7.6	75.0 ± 13.6	74.9 ± 9.4	0.81 ± 0.08
	SVM RBF	81.0 ± 16.7	74.6 ± 7.4	70.9 ± 8.8	78.9 ± 13.7	76.0 ± 8.7	0.81 ± 0.08
	Naive Bayes	79.9 ± 17.1	74.8 ± 8.0	73.1 ± 7.3	78.5 ± 13.7	**76.5** ± **8.9**	**0.81** ± **0.08**
	LDA	78.1 ± 17.5	75.5 ± 7.6	74.6 ± 7.1	77.3 ± 13.7	76.4 ± 9.2	0.81 ± 0.08
	QDA	79.8 ± 17.0	74.7 ± 6.9	73.0 ± 7.4	78.3 ± 13.0	76.3 ± 8.9	0.81 ± 0.08
Single feature: (B) *MF* S2 B3 (3.5-5.0 Hz)	SVM Poly3	86.3 ± 14.8	61.6 ± 4.8	46.1 ± 7.9	77.1 ± 17.1	66.2 ± 7.2	0.68 ± 0.09
	SVM RBF	88.1 ± 13.0	61.2 ± 4.5	44.2 ± 7.5	78.8 ± 16.2	66.2 ± 6.8	0.68 ± 0.09
	Naive Bayes	80.3 ± 16.6	61.6 ± 6.0	49.9 ± 7.5	71.6 ± 17.1	65.1 ± 8.6	0.66 ± 0.11
	LDA	68.5 ± 19.3	62.4 ± 9.1	58.6 ± 7.9	65.1 ± 15.0	63.6 ± 10.1	0.69 ± 0.09
	QDA	80.3 ± 16.5	61.7 ± 6.1	50.1 ± 7.5	71.8 ± 17.1	65.2 ± 8.8	0.66 ± 0.11
Two features (A and B): (A) *PA* S3 B0L’ (0.125-0.575 Hz) (B) *MF* S2 B3 (3.5-5.0 Hz)	SVM Poly3	88.1 ± 12.3	73.3 ± 6.0	67.8 ± 8.1	85.1 ± 11.6	**78.0** ± **7.1**	**0.86** ± **0.07**
	SVM RBF	81.1 ± 16.4	75.5 ± 8.6	73.6 ± 7.8	79.6 ± 12.7	77.4 ± 8.4	0.86 ± 0.07
	Naive Bayes	78.3 ± 16.7	74.9 ± 7.5	73.8 ± 7.9	77.3 ± 13.0	76.0 ± 8.3	0.84 ± 0.08
	LDA	78.0 ± 17.5	75.4 ± 8.6	74.6 ± 6.9	77.2 ± 13.0	76.3 ± 8.9	0.85 ± 0.07
	QDA	77.9 ± 17.3	75.2 ± 7.1	74.4 ± 7.7	77.1 ± 12.6	76.1 ± 8.4	0.83 ± 0.09

The table shows the means and standard deviations for Sensitivity (*Se*), Specificity (*Sp*), Positive Predictive Value (*PPV*), Negative Predictive Value (*NPV*), Classification Accuracy (*CA*), and Area Under the ROC Curve (*AUC*) with *N* = 200 repetitions. For the best single-feature set (A), the highest *CA* and associated *AUC* are in bold and shaded in gray, and the next highest *CA* and associated *AUC* are only shaded in gray. For the two-feature set (A and B), the highest *CA* and associated *AUC* are in bold and shaded in gray. SVM Poly3—Support Vector Machine with third order polynomial kernel, SVM RBF—Support Vector Machine with Radial Basis Function kernel, LDA—Linear Discriminant Analysis, QDA—Quadratic Discriminant Analysis.

### The average decision boundaries

Fig 3 shows the average ROC curves for classification between later premature records (group PL) and later term spontaneous records (group TL), for the best three performing classifiers, i.e. Naive Bayes (shown in cyan), LDA (shown in magenta), and SVM Poly3 (shown in black) using (a) only the best performing single feature *PA* S3 B0L’, (b) only the second best performing feature *MF* S2 B3, and (c) using both features (*PA* + *MF*), as well as (d) the average decision boundaries of these classifiers, according to the results in Table 5. The associated error bars on the ROC curve plots show the standard deviations of the sensitivities (true positive rates) at different false positive rates. Notice how the ROC curves for the best performing single-feature model using only *PA* in Fig 3(a) and for the two-feature model using *PA* + *MF* in Fig 3(c) are very close together, indicating only a slight improvement if using both features.

The average decision boundaries in Fig 3(d) are shown along the scatter plot of feature values in the two-dimensional feature space (values for *PA* S3 B0L’ on the abscissa, and *MF* S2 B3 on the ordinate). The histograms on the top and right sides of the scatter plot show the normalized distribution of values of features *PA* and *MF*, respectively, for premature records (in red) and term records (in green). The red and green dotted lines on the histograms show the fit to the normal distribution according to the feature values for premature and term records, respectively. Note that in premature records, the value of *PA* is higher (top histogram), and the *MF* is lower (right histogram), than in term records. Furthermore, note how the term records are more clustered towards the left, and premature records more towards the right side of the scatter plot. The brown vertical decision boundary line indicates the average decision threshold using only the most promising feature *PA* S3 B0L’ (averaged across all classifiers), and is approximately 0.66. On average, if the value of this feature is above the threshold, the record would be classified as premature, otherwise as term. The average decision boundary for classification using only the second best performing feature, *MF* S2 B3, is not shown since the related classification performance using this feature was inferior, compared to using only *PA*. If using two features, the average decision boundary between premature and term records depends on the classifier, yet for all classifiers the decision boundaries delineate the region of premature records in the lower-right side of the visualized two-dimensional feature space. For classification using the LDA classifier, the decision boundary is a simple line, diagonally splitting the feature space into two regions. For the Naive Bayes and SVM Poly3 classifiers, their decision boundaries resemble a quadratic function and a third degree polynomial function, respectively. Note how the SVM Poly3 decision boundary extends further into the region with higher density of term records, resulting in higher *Se*, but lower *Sp*, compared to the decision boundary of the Naive Bayes classifier.

Fig 4 shows the average ROC curves for classification between later premature (group PL) and later term spontaneous, induced, cesarean, and induced-cesarean (groups TL, IL, CL, and ICL), for the best three performing classifiers, i.e. Naive Bayes (cyan), LDA (magenta), and SVM Poly3 (black) using (a) only the best performing single feature *PA* S3 B0L’, (b) only the second best performing feature *MF* S2 B3, and (c) using both features (*PA* + *MF*), as well as (d) the average decision boundaries of these classifiers, according to the results in Table 6. The ROC curves for these classifiers are quite similar to those shown in Fig 3(a)–3(c), yet for the two-feature models (*PA* + *MF*) they are slightly higher than the ROC curves for classification using only later premature and later term spontaneous records. Furthermore, the error bars, which show the associated standard deviations of sensitivities (true positive rates) at different false positive rates, are more dense than those shown in Fig 3. This is because term pregnancies are represented by more records, resulting in more diversity in the *N* = 200 test sets used for the final classifier evaluation.

Finally, Fig 4(d) shows the associated average decision boundaries for the same three classifiers and the same two-dimensional feature space as also shown in Fig 3(d). However, note that there are many more term records (green) since the term class is now represented with additional records from pregnancies ending in induced, cesarean, and induced-cesarean delivery modes. Furthermore, note the similarity between the decision boundaries shown in Fig 4(d), and the decision boundaries shown in Fig 3(d). In fact, for the single-feature model using only feature *PA* S3 B0L’, the decision threshold, averaged across all classifiers, again equals approximately 0.66, and is visualized with the solid brown vertical line in Fig 4(d).

## Discussion

Methods for the prediction of premature birth based on the EHG increasingly rely on complex sets of features and “black-box” approaches utilizing deep learning. These methods are usually not easily explainable. This is either because it is not easy to explain how the complex interplay of tens or even hundreds of features, each reflective of one or many physiological mechanisms, contributes to the prediction of premature birth, or because the input signal may be transformed into an unfamiliar latent space which lacks any apparent correlation to the underlying physiological mechanisms. Additionally, to our knowledge, no studies employing deep learning approaches for the prediction of premature birth based on the EHG used any of the commonly used interpretability techniques [101] to explain the model’s decisions. Furthermore, if difficult to understand features are used, it can become increasingly difficult to ascribe the feature to any particular physiological mechanism. Even though these contemporary methods may achieve higher classification performance than simpler models, the lack of explainability may prevent their use in clinical practice.

In contrast to such “black-box” approaches, in the present work we sought to find an efficient biomarker, i.e. a single easily derivable and understandable feature, or a combination of at most two such features, with an easily understandable decision boundary for classification between premature and term EHG records. Furthermore, we assessed, for the first time, whether non-spontaneous term delivery modes affect the predictability of premature birth. In this study, we used fully compatible EHG records, collected around the 31st week of gestation, from the currently available datasets TPEHG DB, TPEHGT DS, and ICEHG DS. For feature extraction, we used whole EHG signals, therefore, prior annotation of intervals was not required and the proposed method is fully automated.

### Peak amplitude of the normalized power spectrum in the FWL band

According to the results shown in Fig 1, the most promising feature for classification between later premature (group PL) and later term spontaneous (group TL) records is *PA*, extracted from the horizontally oriented signal S3 in the frequency band B0L’ (0.125-0.575 Hz). These results are consistent with the results of our previous characterization study [81], which showed that feature *PA* S3 B0L’ exhibited the highest power, in terms of the *p*-values, to separate between later premature and later term records. Additionally, the results of the present study (Fig 1) show that *PA* S3 B0L’ is well suited for use with various traditional classifiers, since it always took the first place among the selected features, regardless of which classifier was used.

The band used for extraction of the most promising feature, *PA* S3 B0L’, is mainly reflective of the FWL [81], and its lower and upper bounds almost exactly correspond to the bounds of the FWL band (0.1-0.6 Hz) as defined in [49]. The findings in [81] suggest that the frequency components in the FWL band may be more important than the frequency components in the FWH band for the prediction of premature birth using later recorded EHG records, according to the *p*-values for features extracted from various bands related to the FWL and FWH. Again, the results of the present study are in agreement with those findings, since features extracted from the frequency band B0H’ (0.575-1.0 Hz), which was chosen specifically for its relation to the FWH, were never identified as the first, nor as the second, most promising feature for classification between later premature and later term spontaneous records. This confirms that the frequency components related to the FWL, including those below 0.34 Hz, which are commonly excluded from analysis because of overlap with frequency components due to maternal breathing [88], are important for premature birth prediction.

For comparison of our classification performance results to those of studies related to the prediction of premature birth, we used the *AUC* score, as it is also one of the most commonly reported performance metric for methods based on chemical and molecular biomarkers (Table 1) as well as for methods based on the EHG (Tables 2 and 3). According to the results in Table 5, if using only the most promising feature, *PA* S3 B0L’, for classification between later premature and term spontaneous records, the average *AUC* score is 0.81 ± 0.08, regardless of the used classifier. For comparison to the related methods based on the EHG, we also calculated other widely reported performance metrics *CA*, *Se*, *Sp*, *PPV*, and *NPV*. For the most promising feature, *PA* S3 B0L’, the results in Table 5 show there are slight differences between the performances of the selected classifiers in terms of *CA*, *Se*, *Sp*, *PPV*, and *NPV*. In particular, the best results were achieved using the LDA and Naive Bases classifiers, which yielded the average *CA* of 75.1% ± 9.1% and 74.6% ± 9.3%, respectively, when using later premature and later term spontaneous records. The lowest *CA* was achieved using the SVM Poly3 classifier, as the training algorithm settled, on average, at a less optimal threshold favoring higher *Sp* and *NPV* at the cost of lower *Se* and *PPV*, respectively.

These results suggest that the fully automated non-invasive prediction of premature birth using a single feature, *PA* S3 B0L’, extracted from the EHG collected around the 31st week of gestation, outperforms most recent methods for non-invasive prediction of premature birth based on chemical and molecular biomarkers shown in Table 1. In particular, all methods based on a single chemical or molecular biomarker (upper half of Table 1), yielded lower *AUC* scores (0.79 or less). More strikingly, using only *PA* S3 B0L’ (*AUC* of 0.81) outperforms even most methods based on multiple chemical and molecular biomarkers (lower half of Table 1). The only exception seems to be the method based on expression of multiple genes [40] that yielded only a slightly higher *AUC* of 0.82. As such, in comparison to chemical or molecular biomarkers, the prediction of premature birth using *PA* S3 B0L’ achieves better, or comparable, classification performance in terms of the *AUC* score, yet does not require any potentially costly sample analysis.

For comparison to related studies in the area of premature birth prediction based on the EHG, note that there is no consensus among researchers regarding a benchmark EHG dataset and signal scope (either whole signals, or annotated contraction or dummy intervals). In particular, note that none of the studies in Table 2 used a combined dataset with records of the TPEHG DB and TPEHGT DS to increase the total number of available premature samples for a more realistic assessment of classification performance. Furthermore, most studies used annotated contraction and dummy intervals instead of whole signals. Nevertheless, our results show that *PA* S3 B0L’ outperforms, in terms of *CA* and *AUC*, all but one of the single-feature methods in Table 2. The only exception seems to be the method reported in [73], which yielded a higher *CA* of 84.0% and *AUC* of 0.86 when using *SE* extracted from signal S2 in the band B2 (2.2-3.5 Hz). In that study, the best results were achieved when classifying between annotated dummy intervals in premature and term records of the TPEHGT DS. However, one has to consider that the TPEHGT DS comprises only 13 premature and 13 term EHG records, and on average each record contains about four contraction and four dummy intervals [57]. As such, the intervals from the same record are correlated, yet they may simultaneously end up in both the training and the testing set, which may have resulted in data leakage and overly optimistic results. Nevertheless, in our study we also included *SE* S2 B2 in two feature sets, yet it never took the first place among the selected features (Fig 1). Among the features taking the second place, it was even less frequently selected than *MF* S2 B3 (3.5-5.0 Hz). These results suggest that *SE* S2 B2 is not so promising when using a larger dataset for evaluation of the classification performance.

Comparison to the results of methods based on the EHG using multiple features in Table 3 is somewhat more tentative. Apart from the fact that most studies used different datasets for evaluation, some of the methods may suffer from potential methodological flaws related to feature selection using the whole dataset, improper use of under-sampling without repetitions, or the use of correlated intervals that supposedly represent independent samples. Nevertheless, the results in Table 5 show that the use of *PA* S3 B0L’ alone outperforms several methods listed in Table 3. However, it is important to note that only two of the studies [67, 80] in Table 3 used later records of the TPEHG DB and TPEHGT DS. Moreover, in [67] the authors used a test set comprising overlapping two minute intervals extracted from only four term and four premature records. However, they used only 17 out of 119 later term records of the TPEHG DB, and two later premature records from the TPEHG DB were excluded. As such, their results (*CA* 88.52% and *AUC* 0.89) may be overly optimistic. In contrast, in [80] the authors used all later records from the TPEHG DB and TPEHGT DS and analyzed whole signals, just like in our study. However, despite using a complex deep learning model with over 600 features and obstetric parameters, their method yielded an *AUC* score of only 0.78 (*CA* was not reported). Notably, another study [79] which used a deep learning approach reported an even lower *AUC* score of 0.58, using only records of the TPEHG DB. Interestingly, the use of a single feature, *PA* S3 B0L’ (*AUC* 0.81), outperforms both of these deep learning models.

Furthermore, our method does not perform any complex steps like prior segmentation of EHG signals to remove corrupt signals or specific non-physiological segments. In fact, we intentionally used whole records which leads to efficient and fully automated analysis. Besides, prior segmentation is normally performed in order to further improve the predictive accuracy of the model. For example, in [72] the authors relied on prior manual segmentation by two experts to remove corrupt signal segments to improve the accuracy of the model. Their results suggest that prior segmentation of signals might even improve the classification performance of the models in this study, but unfortunately at the cost of an additional complex pre-processing step. Furthermore, we are aware that the variability of EHG signals may effect the predictive performance of the model, however, the number of available EHG records is too small to perform a thorough analysis.

Nevertheless, using tens or even hundreds of features and deep learning approaches may achieve better classification performance (Table 3). Moreover, deep learning models may also obviate the need for feature engineering, and provided sufficient effort is put into their explainability, they may reveal additional relations between the underlying physiological mechanisms that may contribute to premature birth. However, obstetricians may find it favourable to use a single, easily derivable feature, powering a simple classification model requiring no complex processing steps, to quickly and effectively assess the danger of premature birth. Furthermore, in contrast to more complex models, a single feature may be more easily attributed to known underlying physiological mechanisms. As such, it provides a more effective and direct insight into the physiological changes leading to premature birth. In particular, feature *PA* S3 B0L’ estimates the proportion of the spectral peak amplitude [57] in the frequency band related to the FWL [81], which is believed to reflect the propagation of the electrical activity along the uterus [47, 49, 102]. Several studies have reported that the spectrum of uterine bursts shifts toward higher frequencies as labor approaches [46–48]. The trends reported in [81] suggest that in premature pregnancies, the relative power of the spectrum in the FWL band increases between the 23rd and 31st week of gestation, and this change results in significantly higher values of *PA* S3 B0L’ by the 31st week of gestation in premature pregnancies. The high classification power of *PA* S3 B0L’ (Table 5) suggests that the high relative intensity of the spectral components in the FWL frequency band is an important and effective predictor of premature birth.

Furthermore, when using a single feature for the prediction of premature birth, the decision boundary between premature and term records is simply represented by a single threshold, which may be decreased or increased to move along the ROC curve shown in Fig 3(a). The task of the classifier is then to simply threshold the value of a single feature. However, as evident from the differences in *Se* and *Sp* in Table 5, each classifier yielded a slightly different threshold due to differences in the training algorithms. To avoid bias towards any particular classifier and related training algorithm, we averaged the threshold of the single-feature models across all classifiers, resulting in a single average threshold value for assessing the danger of premature birth. For *PA* S3 B0L’, the average threshold, or decision boundary, is approximately 0.66, and is shown with a solid brown vertical line in Fig 3(d). If the value of *PA* S3 B0L’ is higher than this threshold, this indicates an increased danger of premature birth.

### Median frequency in the band B3

According to the results in Fig 1, the second most promising feature is *MF*, extracted from the vertically oriented signal S2 in the frequency band B3 (3.5-5.0 Hz). Additional, less promising features that took the second place in the histogram in Fig 1(b) include *SE* S2 B2, *PA* S2 B3, and *SE* S2 B0b. Notably, all of these features were extracted from the frequency bands that include the frequency components above 1.0 Hz. Among these features, those extracted from bands B2 and B3, reflective of the influence of the maternal heart on the uterus in terms of the second and third harmonics of the maternal heart rate (*MHR*) seem to be particularly promising. This is in agreement with the results in [57, 73, 81], which also showed the importance of frequency components above 1.0 Hz. According to [57, 81], the *MF* in the band B1 (1.0-2.2 Hz) is believed to be reflective of the *MHR*, and similarly, B2 (2.2-3.5 Hz) and B3 (3.5-5.0 Hz) are believed to be reflective of two times the *MHR* or three times the *MHR*, respectively. Interestingly, among the features extracted from signals in the frequency bands above 1.0 Hz, *MF* S2 B3 yielded the lowest *p*-value to separate between later premature and term records [81]. This is most likely because in band B3 there is less overlap between the frequency components related to the FWH and frequency components related to the maternal heart than in bands B1 and B2. Although many features extracted from signals in the frequency bands below 1.0 Hz exhibited lower *p*-values [81], in our study none of them were included among the most promising features, apart from *PA* S3 B0L’ (Fig 1). This is most likely because those features are correlated (redundant information) to *PA* S3 B0L’, thus, their addition to the feature set did not improve the classification performance. Additionally, the modeled trends of *MF* S2 B3 in [81] suggest that in premature pregnancies *MHR* decreases between the 23rd and 31st week of gestation, but not in term pregnancies. Consequently, the importance of *MF* S2 B3 for the prediction of premature birth, according to the results in Table 1, suggests that this possible relation between premature birth and this decrease of the *MHR* deserves further study.

Nevertheless, if using only *MF* S2 B3 for classification between later premature (group PL) and later term spontaneous (group TL) records, the results in Table 5 show that this feature alone performs poorly, yielding an *AUC* of 0.71 or less. However, when *PA* S3 B0L’ is used together with *MF* S2 B3, the two-feature model using the SVM Poly3 classifier yielded the *AUC* of 0.85 ± 0.07 and *CA* of 76.3% ± 7.8% (lower part of Table 5). Thus, using only two features extracted from signals in non-overlapping frequency bands, each believed to be reflective of distinct underlying physiological mechanisms, resulted in the highest classification performance. The resulting two-feature model represents a slight improvement over the single-feature model that uses only *PA* S3 B0L’. Correspondingly, the ROC curves shown in Fig 3(a) are fairly close to those shown in Fig 3(c).

Most noteworthy, our two-feature model (*PA* S3 B0L’ and *MF* S2 B3) seems to outperform all of the chemical and molecular biomarkers in Table 1, in terms of the *AUC*. On the other hand, very few related studies based on the EHG used only two features (Table 3). In particular, in [73] the authors achieved the highest *CA* of 85.8% and *AUC* of 0.89 using *SE* in the band B2 (2.2-3.5 Hz) for signals S2 (feature 1) and S3 (feature 2), and in [57] the highest *CA* of 70.75% and *AUC* of 0.80 using *PA* in the band B1 (1.0-2.2 Hz) for signal S2 (feature 1) and the tocogram (feature 2). However, both studies used annotated dummy intervals from only 13 premature and 13 term records of the TPEHGT DS. As explained in the previous subsection, the evaluation of classification performance using such a small dataset and correlated intervals may be less realistic.

Additionally, in contrast to more complex models in Table 3 which rely on more than two features, the decision boundaries of our two-feature models (*PA* and *MF*) can still be very simply and effectively visualized. Fig 3(d) shows the decision boundaries, averaged across all (*N* = 200) evaluation runs, when using the SVM Poly3 (dashed blue curve), Naive Bayes (dotted blue curve), and LDA (solid blue line) classifiers. Note how most premature records are located in the lower right corner, “inside” the areas outlined by the average decision boundaries for these three classifiers (higher *PA* S3 B0L’ and lower *MF* S2 B3). This type of visualization makes it fairly easy to observe the differences, in terms of *Se* and *Sp*, between the individual classifiers according to the results Table 5. Although all classifiers using both features exhibit very similar *CA* and *AUC*, the LDA classifier exhibits the best balance between *Se* and *Sp* (and between *PPV* and *NPV*).

In addition, we discuss an example of a simple composite biomarker for assessing the danger of premature birth using only the weighted sum of the two feature values according to the linear decision boundary (diagonal blue line) in Fig 4(d) corresponding to a linear discriminant function:

$$\begin{array}{c} y=\text{sign}(x^{\text{T}}w-b)\;, \end{array}$$ (14)

where *x* = [*PA* S3 B0L’, *MF* S2 B3]^T^, *w* = [5.98, -4.02]^T^, and *b* = -12.78. Hence, if (*x*^T^*w* − *b*) is greater than 0, this indicates danger of premature birth, and the higher the value the higher the probability of premature delivery. This way, the predicted pregnancy outcome (premature/term) can be displayed as a mark (point) in a two dimensional diagram (ordinate—maternal resting heart rate, abscissa—peak amplitude of the normalized power spectrum in the FWL frequency band) displaying also the decision boundary line.

Implementing the predictive model into existing clinical systems would most likely start as an additional clinical investigation, probably on an elective basis at first. Later, if high correlation between the model prediction and premature birth were to be validated by a clinical trial, it can also become routine. A potential challenge would arguably be the initial acceptance by obstetricians, which would need to be convinced by the ease of use and its effectiveness, as well as garnering prior support from medical device manufacturers to support the subsequent development and take further steps towards regulatory approvals. Generally, the use of the proposed model in clinical practice would entail making a non-invasive 30-minute EHG recording around the 31rd week of gestation using a suitable recording apparatus connected to a personal computer, followed by automated digital signal pre-processing, classification, and presentation of the results. The associated direct costs are also expected to be low. The main consumables include five disposable Ag2 Cl electrodes (four placed symmetrically above and below the navel, and one reference electrode placed on the left thigh [82]). The electrodes would then be connected to a personal computer with a high-resolution multi-channel analog-to-digital converter. Due to the low computational complexity of the proposed method, no special hardware processing resources would be required.

The model would be integrated on the connected personal computer, subject to prior regulatory approval of such a diagnostic system for assessing the danger of premature birth. The obtained results using such a system could initially help obstetricians decide on further diagnostic procedures, if the pregnancy is identified at risk for premature birth. This could include more frequent ultrasounds, electronic fetal monitoring, and additional tests related to known risk factors, which would lead to more targeted management of threatened premature birth. Another approach for dealing with risk stratification is using regression analysis (i.e. to predict the pregnancy duration), or treated as a multi-class problem to stratify the EHG recording into sub-categories like extremely premature, very premature, moderate to late premature, knowing the risks for neonatal morbidity and mortality increase with the degree of prematurity [1, 51]. However, this represents a much harder problem than the proposed binary classification (premature vs term) for assessing whether the pregnancy is at risk of premature birth or not, and currently the small number of available premature EHG records would likely make the results of such risk stratification studies unreliable.

### Impact of non-spontaneous delivery modes

For realistic evaluation of methods for the prediction of premature birth based on the EHG, it is important to also consider other common non-spontaneous delivery modes. At the time of recording, it is generally not known whether the pregnancy will end in delivery modes like induced labor or cesarean section. However, to our knowledge, currently existing methods for the prediction of premature birth using the EHG are based solely on classification between EHG records from pregnancies that ended in premature spontaneous or term spontaneous delivery modes, and therefore disregard the possibility of other common delivery modes.

To this end, we used the records of the ICEHG DS, alongside records of the TPEHG DB and TPEHGT DS, to additionally represent the term class with common non-spontaneous delivery modes (i.e. with groups TL, IL, CL, and ICL instead of only group TL). According to the results in Fig 2, the same two most promising features were identified, *PA* S3 B0L’ and *MF* S2 B3, as when only group TL was used to represent the term class (Fig 1). According to the results in Table 6, if using only the most promising feature (*PA* S3 B0L’) for classification between later premature and all later term records, the average *AUC* score is 0.81 ± 0.08, regardless of the used classifier. In terms of *CA*, the best results were achieved using Naive Bayes (*CA* of 76.5% ± 8.9%) and LDA classifiers (*CA* of 76.4% ± 9.2%). If using both of the most promising features (*PA* S3 B0L’ and *MF* S2 B3), the best results were achieved using the SVM Poly3 classifier, yielding the highest *CA* (78.0% ± 7.1%) and *AUC* (0.86 ± 0.07). Since the same two most promising features were identified, these results can be readily compared to the results in Table 5. Notably, the addition of EHG records of the ICEHG DS (groups IL, CL, and ICL) to the term class resulted in even slightly better classification performances when using *PA* S3 B0L’ alone, or when using both features. Furthermore, as shown in Fig 4(d), most non-spontaneous term delivery records also fall “outside” the premature regions outlined by the decision boundary lines. This demonstrates the robustness of the features *PA* S3 B0L’ and *MF* S2 B3 for the prediction of premature birth, regardless of the ultimate term delivery mode, and improves the generalizability of our findings. More importantly, these results show, for the first time, that term non-spontaneous delivery modes appear to match the term spontaneous delivery mode, and do not significantly impact the predictability of premature birth using the EHG.

## Conclusion

In this study, we classified, for the first time, later records of the recently introduced ICEHG DS alongside later records of the TPEHG DB and TPEHGT DS using a variety of standard classifiers. The enrichment of the term class with records from pregnancies ending in other common term non-spontaneous delivery modes enabled us to perform a more robust and realistic evaluation of the classification performance, and assess the impact of these delivery modes on the predictability of premature birth.

In contrast to many contemporary models that use tens and even hundreds of features or complex “black-box” approaches utilizing deep learning, we used at most two easily derivable and explainable features to classify between later premature and term records. To address the premature and term class imbalance and avoid data leakage, we strictly adhered to the recommended Partition-Synthesis evaluation technique. Two of the most promising features, or biomarkers, were identified using feature selection based on the sequential forward selection method, stabilized with the frequency-based aggregation of selected features technique. Using this approach, *PA* S3 B0L’ and *MF* S2 B3 were identified as the most promising features to classify between later premature and term EHG records. Both features are easily derivable, and both are believed to be reflective of distinct physiological mechanisms. The results of this study confirm that the frequency components in the frequency band B0L’, related to the known FWL, as well as the frequency components above 1.0 Hz, related to the influence of the maternal heart on the uterus, are important for the prediction of premature birth.

Furthermore, the identification of the most promising features and the classification performance of the proposed method was not significantly impacted by the inclusion of EHG records from common term non-spontaneous delivery modes. The proposed models, using only the most promising single feature (*PA* S3 B0L’), or using both features (*PA* S3 B0L’ and *MF* S2 B3), appear to outperform the currently existing chemical and biomolecular markers for the prediction of premature birth, as well as many methods for the prediction of premature birth based on the EHG. However, it is important to acknowledge that all currently available EHG datasets contain a relatively modest number of premature EHG records. To validate the classification performance, not only for the methods proposed in this study, but also for other related studies, the results should be confirmed using a consistent evaluation methodology and a larger dataset with a broad range of demographic and clinical characteristics. Moreover, a larger sample size would be very welcome, and would greatly enhance the statistical power and reliability of the results, as well as enable a thorough analysis of the variability in EHG signals and to study how the predictive accuracy of the model is impacted by these variations.

At present, all existing methods for the prediction of premature birth based on the EHG are still in the research phase. Ultimate utilization in clinical practice would necessitate further prospective studies to evaluate the performance of the proposed models in real-time clinical settings and provide stronger evidence of their utility and reliability in practical scenarios. And as for any other existing method for the prediction of premature birth based on the EHG, a suitable clinical trial would need to be designed and implemented to test the method in diverse clinical environments, and report on the feasibility, accuracy, and impact of the model when integrated into standard clinical workflows. This would most likely also include the identification and control for potential confounding factors that could influence EHG signals and the risk of premature birth, which may include maternal health conditions, medications, lifestyle factors, as well other relevant variables identified during post-marketing surveillance.

Additionally, nowadays methods for the prediction of premature birth are becoming increasingly more complex and hardly explainable. However, our results show that *PA* may be used for effective, non-invasive, and fully automated prediction of premature birth, which may outperform even the most recent deep learning approaches. Most importantly, in contrast to those approaches, the inner workings of the models proposed in this study are easily explainable by visualizing the simple two-dimensional feature space and the average decision boundaries of the used classifiers. Moreover, the proposed method is computationally efficient and no special hardware is required for processing the data. All this would simplify integration into existing clinical systems.

In conclusion, we hope that our findings demonstrate the advantages of using simpler models, which can be more easily understood. Furthermore, we hope that our results will provide additional insights into the underlying physiological mechanisms of premature birth and its pathology. Our future work will include efforts to develop a non-invasive, fully automated method for early prediction of delivery mode. This will entail addressing it as a novel multi-class problem encompassing premature and term spontaneous, induced, cesarean, and induced-cesarean delivery modes.
